# Do patents of academic funded researchers enjoy a longer life? A study of patent renewal decisions

**DOI:** 10.1371/journal.pone.0202643

**Published:** 2018-08-29

**Authors:** Leila Tahmooresnejad, Catherine Beaudry

**Affiliations:** Department of Mathematics and Industrial Engineering, Polytechnique Montreal, Montreal, QC, Canada; Universitat Jaume I, SPAIN

## Abstract

This paper assesses the extent to which patent renewal data is associated with government funding in a university context by focusing on the relationship between the funded patentees and renewal decisions of their patents. The aim of this paper is to show whether receiving funding from government contributes to high-value patents as measured by the patent renewal decisions made by their owners. Our observations of academic nanotechnology patents in Canada discovered a positive relationship between funded researchers and the rate of patent renewal after 4 years. Further analysis is also undertaken into the relative impact on patent renewal after 8 years and 12 years. Our results suggest that the length of patent renewal in numbers of years can be related to levels of government funding received by their inventors.

## Introduction

The increasingly expensive patent maintenance fees needed to protect intellectual property bring the necessity to better approximate patent value and to identify relevant indicators for this patent value. Prior studies on the measurement of patent value assume that this value can be reflected in the length of patent renewals [1–7]. These have showed that patent renewal and expiration data are useful indicators to estimate the value of protected innovations and are counted amongst the few sources of established measures of patent value. Renewal decisions reveal the commercial potential that these patents may bring to the market and are an indication that their value exceeds the cost of their maintenance. While patent protection is an important source of economic returns from innovative activities, valuing patents still poses a challenge to policymakers and analysts in science and technology.

Unlike the attention given to industry patenting, the value of university patenting has received less attention in the literature. Moreover, there is a lack of evidence regarding the relationship between research funding and the economic value of patents. After the passage of the Bayh–Dole Act in 1980, university researchers became more active in patenting. The Bayh-Dole Act has been widely cited as an important technology policy that facilitates the patenting and licensing of academic-led and federally-funded inventions in the US. This act enabled universities to retain the rights to patents which emanate from federally funded research programs and encouraged them to collaborate with industry to promote the utilization of their patents arising from such public funding. The passage of this legislation changed the way academic inventors develop technologies from government-funded research in the US and since then, many countries have adopted similar policies [8–10].

The nature of funding behind university inventions could provide a useful platform though which to study their commercialization and transformation into new products. Although inventions that have been developed using industry funding are intuitively more likely to result in commercial success, because they are often the result of a request or need from the private sector, we hypothesize that government-funded patents owned by universities could also be associated with higher patent value compared with those of less funded or not funded academic inventors. We shed light on this question by studying under which conditions university nanotechnology patents are more valuable, i.e. when it is worth paying the patent renewal fees. Nanotechnology has been under scrutiny ever since its emergence and has led to many patents. Yet, it is still very much a science-based field and as such provides a perfect case for our analysis of the factors that influence university patent renewals.

Because of the substantial public investment in nanotechnology research, the economic value of the resulting academic patents, as a measure of impact of these policies, is of great importance to policy makers. Public decision makers generally combine various patent indicators to measure the impact of their policies on technological outputs in order to infer economic development or performance. The recent CCA report in 2018 [11] on the state of science and technology (S&T) and industrial R&D is the perfect example of this type of measurement. Despite the fact that patent counts are commonly used for this purpose, mainly due to a lack of data on other measures, it is accepted that alternatives related to the importance or value are more useful and give more reliable and practical results. For example Carpenter et al. [12] worked on backward citations, Lerner [13] used the scope of patents, Tong and Frame [14] applied the claims and Lanjouw [15] employed the renewal of patents as value indicators.

The article focuses on the relationship between government support for university research and commercial potential of patents exemplified by renewal decisions by academics and/or their universities. It provides evidence to the effect that receiving government funding infers a higher economic value of academic innovations and as such constitutes a novel research avenue that differs from prior studies of patent renewal data. We are fortunate to have access to a full database of Canadian federal funding with which to explore this relationship. For this purpose, we employ a sample of Canadian patenting data extracted from the United States Patent and Trademark Office (USPTO) integrated with Canadian federal funding agencies data.

The economic literature suggests that characteristics such as including more claims, having more backward citations, and more frequent citations by later patents, constitute evidence of the private value of patents. In this study we also demonstrate the relevance of patent quality indicators–the number of citations that patents received, and the number of claims contained in the patents–on patent renewal decisions. While patent citations are a generally accepted measure of quality that can be used to approximate patent value [16], there is a more complex relationship between the number of patent claims and patent value as it is not systematically associated with patent value [17,18]. Patents with a higher number of claims are better protected and can be more important; yet, the number of claims is an underutilized indicator to measure patent value. One can argue that the number of claims is the most informative measures, but it might be affected by the examiners. Patentees have incentives to claim as much as it is possible to define the rights of the patentees when they file their applications, but the examiners may ask them to narrow the claims. The other factor that would affect the number of claims is the change in patent application fees [17,19].

We find that receiving public funding has a positive effect on the value of patents in universities and these patents are more likely to be renewed in subsequent years. Our analysis establishes clear evidence that government funding is fruitful for researchers in universities and increases the value of the patents to be commercialized in industry.

The remainder of the paper is organized as follows. Section 2 presents the relevant literature to our theoretical framework and our hypotheses surrounding patent renewal decisions. Section 3 provides a detailed description of the dataset and the constructed variables that are used in this study. Section 4 links funding and two patent indicators to patent renewal decisions and presents the results for the empirical testing our hypotheses. Finally, Section 5 concludes with a brief discussion of the results and of their implications.

## Literature review and proposed hypotheses

Most firms that patent are influenced by the potential value of their patents, and patent portfolios are an intrinsic part of the value creation process of a firm. Firms with valuable patent portfolios and strong production capabilities are better positioned to capture greater market share. According to Grimaldi et al. [20], a valuable patent portfolio is essential for a firm’s strategic business objectives and as such supports the value creation process of the firm.

The precise value that a patent contributes to a product is a rather subjective issue and assessing market value of a specific patent in a given product when multiple and complementary patents are incorporated in the said product is a complicated matter [21]. Patents that have higher commercial value can drive technological advances and are of great importance for economists and policymakers. Various indicators have been developed in prior studies on patent statistics—including patent citations, value-weighted patent counts, litigated patents, licensing, and patent renewal—to explore the value estimation of inventions [3]. Pakes and Schankerman [4], Pakes [22], and Schankerman and Pakes [7] were the first to develop models on patent renewal decisions aimed at assessing patent value. Because renewing a patent implies a cost, patent renewals are interpreted as indicators of the expected value of patents. These studies assume that only economically profitable patents are renewed for longer periods of time. Patent renewal decisions also offer a metric of self-valuation of patents by their owners. Using a survey-based estimation of the economic value of patents, Harhoff et al. [23] highlighted that full-term patents are more valuable on average compared to those allowed to lapse at midterm.

To get the full extent of patent protection, owners should pay their maintenance fees three times in a patent’s legal life, and each time, the amount required is larger than for the previous stage. Generally, if the all maintenance fees are paid, patents will expire after 20 years from the granting date. According to United States Patent and Trademark Office (USPTO) policies, if the maintenance fees are not paid by the end of 4^th^, 8^th^ or 12^th^ years after the grant date of a patent, the patent is considered expired and the entire patent rights lapse. Rational holders of patents should only desire to pay additional fees if patent value is high and exceeds the cost of renewal fees [5]. Meyer and Tang [3] indicated that patentees engage in a renewal decision if the estimation of profit return of the patent is greater than the current cost of renewal. Patent holders also probably decide to patent in patent offices of other countries where net returns are positive on the international flow of returns. Lanjouw and Schankerman [24] showed that patent quality, measured by an index of patent citations and number of claims, plays a role in the renewal decisions of patentees.

### Patent indicators

The literature presents a wide range of patent-based measures to characterize the value of an invention. One of the proxies of patent value considered is the citation impact: The number of times that a patent is considered relevant prior art is an indicator of its impact. Prior inventions set the stage for new inventions, and receiving more forward citations shows the potential embodied in these inventions that could stimulate new contributions [4,23,24,25]. Patent citations provide a dynamic view of the technological antecedents and descendants of the patented invention, and thus allows scientists to identify highly cited patents for which the impact on subsequent inventions is the greatest. It is notable that a patent with no forward citations is a technological dead end.

Moreover, the number of forward citations that a patent receives characterizes its importance in advancing technology [26] and has been associated with patent value [23,27,28]. Although a few scholars have expressed skepticism regarding this association [29,30], patent citations remain the foremost indicator of technological impact that has been shown to be correlated with economic value [31]. For instance, Narin et al. [32] found that the average citation frequency of a company’s patent portfolio is associated with the company’s profits. Using data from two major universities (University of California and Colombia University), Sampat and Ziedonis [33] showed that patent citations are only a good revenue predictor for a patent that is licensed. Mogee et al. [34] illustrated that patent citations are positively related to renewal model value estimates. Harhoff et al. [23] concluded that renewed patents receive more citations and have higher economic value. Similarly, Maurseth [25] found citations as one of the factors related to patent renewal. Regarding the particular case of examiner’s citations, Hegde and Sampat [35] noted that these are positively related to the probability of renewal decisions using the 4, 8, and 12 year renewals as an indicator. Another study validating the usefulness of examiners’ forward citations by Yasukawa and Kano [36], confirmed that citations made by examiners to assert the novelty of the invention are a useful indicator of the potential value of patents, and they are more likely to be renewed for a full term.

The prevailing evidence in favor of patent citations as a significant predictor of the value of patents leads us to propose our first hypothesis. H1 therefore aims to verify whether the link between patent citations and renewals is applicable in the Canadian nanotechnology academic context.

***H1***: *Patents with higher number of forward citations within a 5-year period have a higher rate of patent renewal in 4*, *8 and 12 years after their grant year*.

A patent stating a greater number of claims implies a wider range of protection and a potentially greater value of intellectual property to be protected. The correlation between the number of claims included in a patent application and patent value is however still subject to debate. Claims are one of the most important parts of the patent document, as they delineate the boundaries of the intellectual property of the patentee. The interpretation of these claims plays an important role in the legal and economic impact of a patent, in particular in regard to licensing. Whether their numbers is an appropriate indicator is still unclear. Allison et al. [17] highlighted the number of claims as one of the characteristics used by economists to predict the value of patents. Moore [18] showed that there is a correlation between patents which were not maintained the full term and a lower number of claims.

Although the number of claims in granted patents is sensitive to the examination process and examiners may drop or redefine claims, this indicator is considered a meaningful measure of patent quality that captures the technological and economical value of patented innovations [37]. The structure of the patent fees depends on the number of claims, and patents with a large number of claims demand higher fees for their maintenance. This structure reflects the expected market value of the patent and is an indicator of the willingness to pay for broader protection [38]. Moreover, the number of claims listed in the patent documents is affected by the changes that have happened over the years in the claim-related fees structure. In December 2004, when the USPTO invoked a new claims fee structure, introduced in order to obtain a clear definition of the patent scope and to reduce the complexity of patent documents [39], and designed to incentivize patentees to reduce their numbers of claims, the (perceived excess) number of claims per granted patent declined.

Although the literature is not overwhelmingly conclusive regarding whether the number of claims is a good indicator of patent value, there is enough support in the literature to suggest a link with patent quality [24,38,40,41]. For instance, because patents with a higher number of claims are more likely to be involved in litigations processes to protect their intellectual property [19] it is inferred that they have a higher value. We therefore expect to find a positive correlation between the number of patent claims and renewal decisions, which leads us to our second hypothesis:

***H2***: *Patents with a higher number of claims have a higher rate of patent renewal in 4*, *8 and 12 years after their grant year*.

### Funding impact

Most measures of patent value are used in the context of industry, and few studies have analysed university patenting. Academic inventors raise research funds, and the provision of government funding into academia positively affects the development of patents. The question is whether these government-funded inventions actually lead to commercialised innovations. While the link between industry funding and the commercialization of patents is rather intuitive, academic research plays a core role in contributing to basic knowledge via scientific publication. It is indeed assumed that the “return on [research] investment” is not fully appropriable particularly in emerging technologies such as nanotechnology, where uncertainty remains an important issue. According to [42], the main goal of policymakers in financing universities is to encourage their traditional role of performing research and advancing knowledge. Although the United States Bayh-Dole Act in 1980 paved the way for universities and academic inventors to retain the intellectual property of their federally funded patents, mixed evidence is found regarding patent quality despite an overall increase in the number of academic patents [43,44]. Furthermore, university patents owned by academic institutions imply a lower commercial value compared to the academic patents owned by industry [45]. The difference could be related to the fundamental nature of academic-owned patents.

To the best of our knowledge, very few studies have linked government funding to patent renewal decisions, likely due to the lack of data. This relationship is worth exploring and understanding as it can yield important implications for policy design. Because nanotechnology has experienced significant government funding in recent decades, mainly because it is recognized as a critical domain, measuring the value of this technological output is of very high relevance to policy makers. Significant public funding and national programs in various countries have been provided to boost research and the development of technological output for this interdisciplinary field [46,47]. While the literature [48, 49] has found that funded researchers contribute to higher quality patents measured by the number of citations, the impact of funding on the economic value of academic patents has not been clearly identified. Hence, we expect that the positive correlation between funding and patent quality found in the literature should be extended to a positive impact of funding on patent renewals as one of the patent value indicators. To shed light on this issue we suggest the following hypothesis:

***H3***: *Patents associated with government-funded researchers in university have a higher rate of patent renewal in 4*, *8 and 12 years after their grant year*.

## Data and methodology

### Data

The data used in this study comprises government funding for academic research, university patenting, as well as number of citations and claims to examine the relationship between these indicators and patent renewal decisions in the field of nanotechnology. The use of academic patents data enables us to examine the impact of government funding for the purposes of generating future revenues from research.

We extracted patenting data from the United States Patent and Trademark Office (we used the USPTO because the US is the greatest economic partner for Canada, and Canadian inventors mostly protect their inventions in the USPTO as well), regarding names of inventors, their addresses, the number of citations, the number of claims, the application and grant years of patents in addition to information on patent renewals. The data on patent renewal decisions is obtained from patent maintenance fees events registered in the USPTO (maintenance fees events filed in the USPTO contains information from September 1981 to the present).

To specifically extract the Canadian patents, we selected all the inventors who have an address or affiliation in Canada. We integrated information from the Canadian federal funding agencies database to identify whether the academic-inventors of these patents obtained government funding. This funding information database provides yearly grant amounts from the Natural Sciences and Engineering Research Council of Canada (NSERC) and the Canadian Institutes of Health Research (CIHR). (“In Canada, the federal government funds three major research–granting councils: the Natural Sciences and Engineering Research Council, the Canadian Institutes of Health Research, and the Social Sciences and Humanities Research Council. In 2007–08, the budgets of the three councils totaled over $2.1 billion. Sponsored research projects conducted under contract for various federal government departments receive additional federal funding. To leverage Canada's research capabilities in a number of areas of strategic importance, the federal government maintains the Networks of Centers of Excellence, funded by the above-mentioned councils, to link researchers from universities, industry, and governments across the country.” (https://www.cicic.ca). For more information please see http://www.nce-rce.gc.ca and http://www.cihr-irsc.gc.ca).

The Canadian institutes of Health Research and the Natural sciences and engineering research council of Canada are charged with promoting and funding research and research training in Canada to pursue opportunities for generating world-firsts in knowledge, and expanding the frontiers of science and engineering. The institutions which are engaged in research must recognize the importance of knowledge and new knowledge and applications that they create and must make sure the funded research meets the highest standards of excellence. These federal agencies have harmonized their institutional eligibility requirements which is necessary for institutions to promote and assist research and develop and implement effective policies, procedures and controls to make sure that the funds are used as effectively as possible. Moreover, the parties are committed to promoting the responsible conduct of research and ensure that the activities are in accordance with the highest ethical and financial standards (please see http://www.science.gc.ca).

We matched our databases over the time period 1985–2005, but we analyze the data for the period 1996–2005 because too few nanotechnology patents are present during the 1985–1995 period. The year 2005 is chosen as the end year for our sample for three reasons. First, some patents may take more than 5 years to be granted. Second, we considered three time windows for citations: 3-year, 5-year and 7-year forward citations after the patent grant year. Third, to maintain a patent in force after a patent is granted, the patent holder must pay a maintenance fee every 4 years three times after a patent is granted. To observe the third stage renewal, we have to wait 12 years after the grant year i.e. up to 2017.

### Dependent and explanatory variables

We built a panel dataset at the individual academic-inventor level and defined our dependent variables based on a ratio of the total number of patent renewals attributed to an individual academic inventor divided by the total number of patents of that individual inventor (we have data for each individual over a period of 10 years, 1996–2005). Three dependent variables were constructed based on the number of the years that a patent was renewed: to study the factors that influence whether a patent was renewed 4 years after the grant year (*PatentRenew4*), we use Eq 1; for patents renewed after 8 years (*PatentRenew8*), we use Eq 2; and for patent renewed after 12 years (*PatentRenew12*), we use Eq 3 (we use the total number of patents by year in these equations). These three variables measure the proportion of patents that are renewed in a given year with respect to all patents on which an academic is a named inventor. Assignees cannot renew patents in the second phase (after 8 years) if they have not renewed patent protection after 4 years. The same is true for renewal after 12 years, which is deemed the most valuable renewal. We also define three associated dummy variables (*dPatentRenew4*, *dPatentRenew8* and *dPatentRenew12*) to indicate whether patents were renewed or not. These dummy variables take the value 1 if their equivalent proportion variable is different from 0, and are used in probit regressions to estimate the probability of being renewed in subsequent years based on the funding that their inventors received.

$$PatentRenew4=\frac{The\;number\;of\;patents\;of\;an\;individual\;academic\;inventor\;that\;were\;renewed\;4\;years\;after\;their\;grant\;year}{Total\;number\;of\;patents\;for\;that\;individual\;inventor}$$ (Eq 1)

$$PatentRenew8=\frac{The\;number\;of\;patents\;of\;an\;individual\;academic\;inventor\;that\;were\;renewed\;8\;years\;after\;their\;grant\;year}{Total\;number\;of\;patents\;for\;that\;individual\;inventor}$$ (Eq 2)

$$PatentRenew12=\frac{The\;number\;of\;patents\;of\;an\;individual\;academic\;inventor\;that\;were\;renewed\;12\;years\;after\;their\;grant\;year}{Total\;number\;of\;patents\;for\;that\;individual\;inventor}$$ (Eq 3)

Because patents of higher quality are likely to be of greater value (H1), we therefore use the number of forward citations as an explanatory variable to find their relationship with patent renewal decisions. Patents that are renewed are more likely to be highly cited than patents that are allowed to expire before their full term terminates.

We divide the number of forward citations received by the patents granted to an academic inventor over the 5-year period (in the final models, we found that 5-year citations for patents give us more consistently significant results compared to 3-year and 7-year time windows) after the patent grant year by the total number of patents of the same academic inventor (AvgCitPerPat). Because the analysis is performed at the individual academic inventor level, all data were aggregated at the academic inventor-year level to create a panel database.

$$AvgCitPerPat=\frac{Total\;number\;of\;forward\;citations\;of\;all\;patents\;of\;an\;individual\;inventor\;5\;years\;after\;their\;patent\;grant\;year\;}{Total\;number\;of\;patents\;for\;that\;individual\;inventor}$$ (Eq 4)

Similarly, the explanatory variable related to the number of claims to examine our second hypothesis was calculated using the same principle as for the number of citations (*AvgClaimPerPat*).

$$AvgClaimPerPat=\frac{Total\;number\;of\;claims\;for\;all\;patents\;of\;an\;individual\;inventor}{Total\;number\;of\;patents\;for\;their\;individual\;inventor}$$ (Eq 5)

Our third explanatory variable is based on the accumulated amount of funding obtained by academic inventors in each year (*PubFunding*). Government funding has long been recognized as critical to knowledge development in universities. Since the patenting is more likely to happen at the end of a funding period, i.e. after the research has been performed, we include a one-year lag for funding before the patent application to avoid contemporaneous effects of government funding.

Finally, we account for regional fixed effects by defining dummy variables for each province: *dQC* takes value 1 if inventors are from Quebec universities, 0 otherwise; *dON* takes value 1 if inventors are from Ontario universities, 0 otherwise; *dBC* takes value 1 if inventors are from British Columbia universities, 0 otherwise; *dAL* takes value 1 if inventors are from Alberta universities, 0 otherwise; *dCanada_others* takes value 1 if inventors are from universities of provinces other than Quebec, Ontario, British Columbia and Alberta, 0 otherwise.

### Models

Three methods are used in our analysis of the factors that affect patent renewal decisions in 4, 8 and 12 years as dependent variables. First, we examine the impact of funding and patent quality measures on patent renewal ratio using a Tobit model because the dependent variable has an upper boundary of 1. This model, also called a *censored regression model*, was suggested by James Tobin [50] to describe a relationship between dependent and independent variables when there is either right censoring or left censoring in the dependent variable [51].

Second, transforming the ratios of patent renewals into dummy variables for each of these 4-, 8- and 12-year renewals we then use a probit model to estimate the probability of being renewed after 4, 8 and 12 years of a granting patent. Both types of models can be expressed as:

$$\left(\begin{array}{c} PatentRenew4,PatentRenew8,PatentRenew12\\ dPatentRenew4,dPatentRenew8,dPatentRenew12 \end{array}\right)=f\left(\begin{array}{c} PubFunding,\\ AvgCitPerPat,AvgClaimPerPat\\ nbPatCum,\\ dQC,dON,dBC,dAL,dCanada\_others \end{array}\right)$$ (Eq 6)

### Instrumental variables

During the course of this study, we suspected that our models suffer from potential endogeneity of two different kinds. First, there is an intrinsic connection between the research productivity of individual academic inventors and the amount of government funding they received. The probability of obtaining funding depends on past patents and publications which also depend on the funding available prior to patenting and publication activities. This correlation between the funding lags is apparent to that of simultaneity and is a common cause of potential endogeneity. Second, there may be the unobserved heterogeneity due to inherent quality of academic inventors. “Better” or more prolific academic inventors receive more and/or larger grants and this may affect the future amount of funding raised. To correct for this problem, we used instrumental variables and tested whether our funding variable is endogenous.

We include three instruments that explain the unobserved capabilities of academic inventors as higher quality researchers that are more likely to receive greater amounts of public funding. Measuring the potential quality of inventors on the basis of their past record may influence their capacity of raising public funds.

For this reason, we use the type of research chair (*CAResearchChair*) that a researcher occupies at some point in her career by defining a dummy variable, which takes the value 0 for no chair, and the value 1 for holding a Canada Research Chair or an industrial chair and receiving funding from NSERC or CIHR.

We also use the career age (*ResearchCareerAge*) of researchers in the nanotechnology field as a proxy for real age, as older scientists are generally better funded. Finally, we take into account the cumulative number of articles published by these academic inventors (*nbArtCum*), since our focus in this study is on academic inventors and the main activity of academic researchers is publishing the articles. We estimate models such as two-stage least-squares (ivregress), instrumental variable Tobit (ivtobit) and and probit (ivprobit) models to account for the potential endogoneity of our funding variable. We estimate models of the following general form:

$$PubFunding=f\left(\begin{array}{c} AvgCitPerPat,AvgClaimPerPat\\ nbPatCum,\\ dQC,dON,dBC,dAL,dCanada\_others\\ ResearchCareerAge\\ dCAResearchChair,nbArtCum \end{array}\right)$$

$$\left(\begin{array}{c} PatentRenew4,PatentRenew8,PatentRenew12\\ dPatentRenew4,dPatentRenew8,dPatentRenew12 \end{array}\right)=f\left(\begin{array}{c} Predicted_{first\_stage}(PubFunding),\\ AvgCitPerPat,AvgClaimPerPat\\ nbPatCum,\\ dQC,dON,dBC,dAL,dCanada\_others \end{array}\right)$$ (Eq 7)

The Hausman specification test rejected the null hypothesis that our variables are exogenous, which strongly suggests the presence of endogeneity in the models. Further, the Sargan-Hansen test (this is the test of overidentifying restrictions and under the null hypothesis we accept that the instruments are valid) showed that the instruments selected were valid. For the issue regarding instruments relevance, Anderson [52] proposes an approach which considers the canonical correlations. Anderson’s test assumed that the regressors are distributed multivariate normal and if we cannot reject this null hypothesis, the identification status of the estimated equation is unidentified. For the instruments used in this study, the results suggest that the model is identified, i.e. that they are relevant and correlated with the endogenous regressors. These results allow the use of ivtobit and ivprobit to fit tobit and probit models where funding covariates are endogenously determined.

### Patent survival analysis

As an alternative to our analysis at the individual academic inventor level, we also use survival analysis at the patent level, employing Cox proportional hazard model to study how the factors are associated with the “survival or death” of a patent. We assume the death of a patent as the hazard and consider a patent has lapsed, or has “died”, if it was not renewed after 4, 8 or even 12 years. We define the specified time period until the occurrence of the patent’s death as *t*, where *t* takes value 1 if patent was not renewed after 4 years, takes the value 2 if patent was renewed after 4 years but was not renewed after 8 years, takes the value 3 if the patent was renewed two times but not for the third time after 12 years and the value 4 if it was renewed after 12 years and thus survives its entire legal life within the patent system.

For each patent in our sample, we compute the average amount of public funding (*AvgPubFunding*) and a number of control variables including number of assignees (*NbAssignees*), number of inventors (*NbInventors*), number of forward citations (*ForwardCit*), number of backward citations (*BackwardCit*) and number of claims (*NbClaims*).

## Descriptive statistics

**Fig 1:**
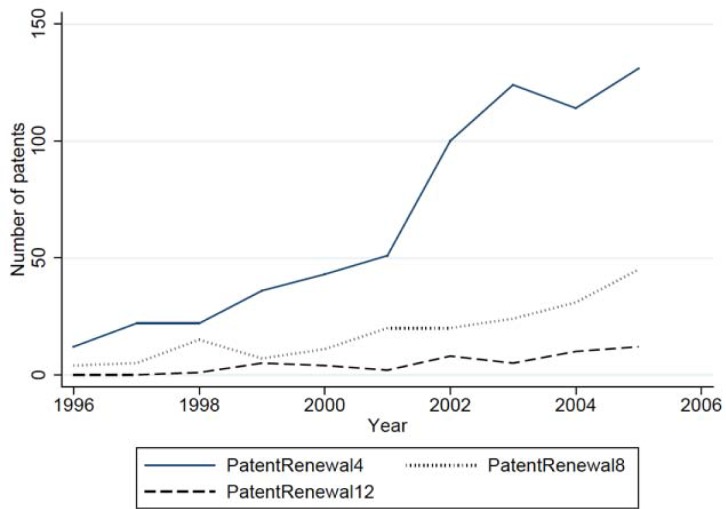
Comparing the patent renewal in 4, 8 and 12 years.

Our sample comprises 7,664 observations, for 1,112 academic inventors, over a period of 10 years. Some scientists had not started their career in 1996 and other were no longer active in 2005, hence the unbalanced panel. The descriptive statistics of the sample are presented in S1 Table (correlation matrix) and S2 Table (descriptive statistics) in supporting information. Fig 1 shows that most of patents are renewed for the first time at year 4, but that the rate of renewal is much lower after 8 and 12 years.

**Fig 2:**
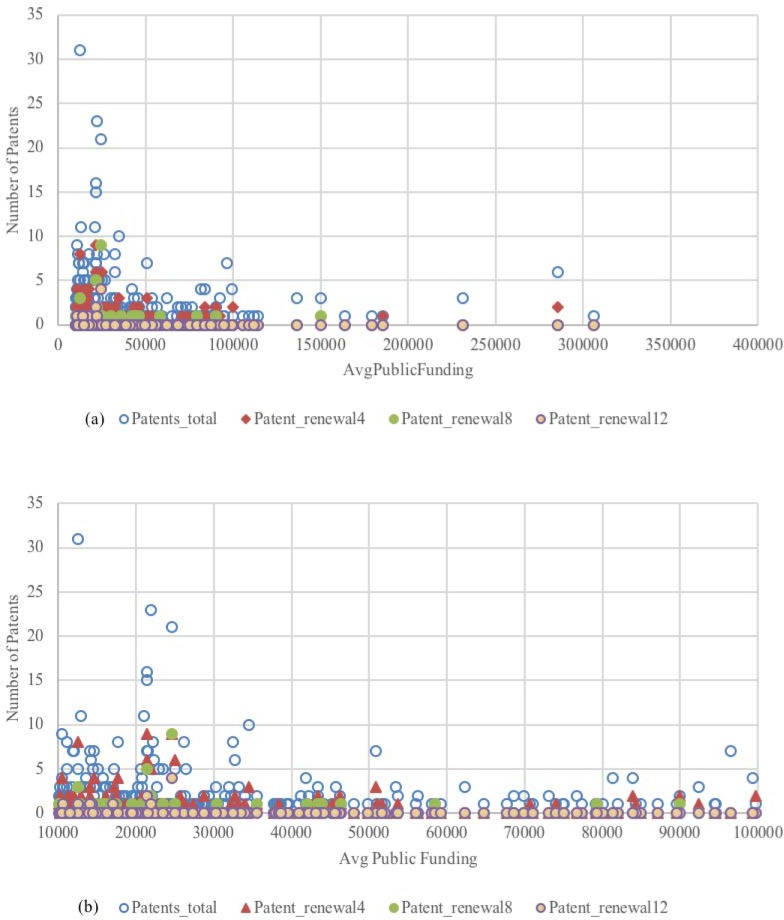
Number of patent renewals by average amount of funding.

Fig 2A and 2B provide an overview of the average number of patents and average number of patent renewals in 4, 8 and 12 years with respect to the average amount of public funding raised by academic inventors. As public funding increases, the number of renewals observed become increasingly low. Funding seem to play a role for the amounts below $120,000. Fig 2B focuses on inventors who received funds between $10,000 and $100,000. While patents are renewed after 4 and 8 years at a relatively high rate, inventors more often let their patent applications lapse after 12 years without requesting a third patent renewal. Furthermore, the proportion of patents that are renewed in subsequent years shows decreasing returns. In our sample, 55% of the inventors have at least one patent over a period 10 year. Overall, 32% of inventors renewed at least one patent after 4 years, whereas 68% of inventors never renewed their patents at all. After 8 years, these values are much lower: 13% of inventors renewed at least one patent; and the observed patent renewal for 12 years is only of 4%.

## Regression results

This section presents the results of the three types of models (ivtobit, ivprobit and Cox proportional hazard regressions) on the patent renewal decisions (results for ordinary least squares and two-stage least squares regressions are presented in S3, S4 and S5 Tables in supporting information). Multiple regression estimations for different specifications of each model were conducted to evaluate the sensitivity of our results. Separate regressions (models 1 to 3) were estimated to evaluate whether the amount of funding received by inventors for their research, the number of citations and the number of claims of their patents influence the value of these patents. Year dummies were added to control unobserved year fixed effects. The Wald chi-square values are highly significant, confirming that our models fit the data significantly better than a null model (we also use the number of renewed patents of an inventor as dependent variable instead of the ratio in our analysis and the results were the same. Please see S6, S7 and S8 Tables in supporting information).

In each of the three tables, Tables 1, 2 and 3, we report both the second stage (top of the table) and the first stage (bottom of the table) regressions for the ivtobit and ivprobit models. In all three tables, we find a consistently positive and significant effect of our three instruments (*dCanadaChair*, *Age* and *nbArtCum*), suggesting that holding a research chair, being older and having published more articles in the past explain part of the quality of academic researchers who succeed in raising public funding. It is not surprising that the cumulative number of articles published by an academic inventor plays a role in receiving a greater amount of funding. Publishing articles provides visibility to researchers and increases their relative citation rate, which both matter when submitting grant proposals. The prestige of the researcher provides a greater visibility for that researcher and highlights their research and development excellence. A research chair demonstrates the ability of the individual in attracting research funding based on the excellence of her research and has the expected positive influence on the likelihood of raising more government funding.

**Table 1:** Impact of government funding on 4-year patent renewal decisions (PatentRenew4) in Canada–Regression results of the ivtobit and ivprobit model.

*Variables*	ivtobit dependent variable: PatentRenew4						ivprobit dependent variable: dPatentRenew4					
	(1)		(2)		(3)		(1)		(2)		(3)	
*ln(PubFunding)*_*t-1*_	0.0805	***	0.1069	***	0.0909	***	0.1014	***	0.1294	***	0.1186	***
	(0.0128)		(0.0139)		(0.0130)		(0.0127)		(0.0108)		(0.0119)	
*ln(nbPatCum)*_*t*_	0.3787	***					0.6614	***				
	(0.0297)						(0.0484)					
*ln (AvgCitPerPat)*_*t*_			0.3454	*					0.6566	**		
			(0.2002)						(0.2834)			
*[ln (AvgCitPerPat)*_*t*_*]*^*2*^			-0.1527						-0.3101	*		
			(0.1267)						(0.1840)			
*ln (AvgClaimPerPat)*_*t*_					0.3494	***					0.5922	***
					(0.0841)						(0.1302)	
*[ln (AvgClaimPerPat)*_*t*_*]*^*2*^					-0.1968	***					-0.3043	***
					(0.0357)						(0.0569)	
*dQC*	0.2760	***	0.3421	***	0.2924	***	0.3444	***	0.4070	***	0.3738	***
	(0.0658)		(0.0754)		(0.0698)		(0.0803)		(0.0831)		(0.0845)	
*dON*	0.2965	***	0.3557	***	0.3106	***	0.3807	***	0.4251	***	0.3994	***
	(0.0624)		(0.0730)		(0.0681)		(0.0721)		(0.0798)		(0.0815)	
*dBC*	0.2040	**	0.3361	***	0.2852	***	0.2433	**	0.4209	***	0.3839	***
	(0.0822)		(0.0982)		(0.0929)		(0.1049)		(0.1173)		(0.1197)	
*dAL*	0.2864	**	0.4276	***	0.3605	***	0.3732	**	0.5358	***	0.4881	***
	(0.1136)		(0.1452)		(0.1354)		(0.1536)		(0.1817)		(0.1846)	
*Constant*	-2.3185	***	-2.1267	***	-1.9205	***	-3.1411	***	-2.5053	***	-2.4243	***
	(0.1836)		(0.1875)		(0.1761)		(0.1199)		(0.1068)		(0.1184)	
***First stage***: ***ln(PubFunding)***_***t-1***_												
*ln(nbPatCum)*_*t*_	-0.3169						-0.3188					
	(0.2243)						(0.2242)					
*ln (AvgCitPerPat)*_*t*_			-0.5212						-0.5329			
			(0.7847)						(0.7862)			
*[ln (AvgCitPerPat)*_*t*_*]*^*2*^			0.0334						0.0371			
			(0.3678)						(0.3682)			
*ln (AvgClaimPerPat)*_*t*_					-0.3969						-0.4024	*
					(0.2425)						(0.2433)	
*[ln (AvgClaimPerPat)*_*t*_*]*^*2*^					0.2121	***					0.2134	***
					(0.0722)						(0.0724)	
*dQC*	-2.2127	***	-2.2738	***	-2.2337	***	-2.2102	***	-2.2869	***	-2.2455	***
	(0.3694)		(0.3703)		(0.3707)		(0.3695)		(0.3708)		(0.3711)	
*dON*	-2.3168	***	-2.3730	***	-2.3473	***	-2.3132	***	-2.3868	***	-2.3596	***
	(0.3623)		(0.3622)		(0.3638)		(0.3625)		(0.3625)		(0.3640)	
*dBC*	-2.5048	***	-2.5958	***	-2.5690	***	-2.5009	***	-2.6120	***	-2.5833	***
	(0.5113)		(0.5086)		(0.5083)		(0.5112)		(0.5088)		(0.5084)	
*dAL*	-3.1797	***	-3.2557	***	-3.2083	***	-3.1780	***	-3.2726	***	-3.2234	***
	(0.7280)		(0.7190)		(0.7182)		(0.7286)		(0.7192)		(0.7184)	
*dCAResearchChair*_*t*_	2.6527	***	2.3139	***	2.4611	***	2.6194	***	2.1870	***	2.3395	***
	(0.4652)		(0.4641)		(0.4696)		(0.4655)		(0.4651)		(0.4717)	
*ResearchCareerAge*_*t*_	0.8780	***	0.8737	***	0.9139	***	0.8700	***	0.8643	***	0.9060	***
	(0.0628)		(0.0602)		(0.0639)		(0.0633)		(0.0608)		(0.0644)	
*[ResearchCarerAge*_*t*_*]*^*2*^	-0.0262	***	-0.0264	***	-0.0277	***	-0.0257	***	-0.0259	***	-0.0273	***
	(0.0028)		(0.0027)		(0.0028)		(0.0029)		(0.0027)		(0.0028)	
*ln(nbArtCum*_*t*_*)*	-0.9736	***	-0.8713	***	-0.9276	***	-0.9599	***	-0.8289	***	-0.8877	***
	(0.2686)		(0.2595)		(0.2678)		(0.2715)		(0.2627)		(0.2705)	
*[ln(nbArtCum*_*t*_*)]*^*2*^	0.2046	***	0.1901	***	0.1994	***	0.2012	***	0.1837	***	0.1929	***
	(0.0726)		(0.0679)		(0.0709)		(0.0735)		(0.0683)		(0.0711)	
*Constant*	5.6030	***	5.3486	***	5.0602	***	5.6131	***	5.3616	***	5.0708	***
	(0.4743)		(0.4644)		(0.4774)		(0.4739)		(0.4657)		(0.4787)	
*ln(α)*	-0.0785	***	-0.1075	***	-0.0903	***						
	(0.0129)		(0.0139)		(0.0131)							
*Nb observations*	7664		7664		7664		7664		7664		7664	
*Wald χ*^*2*^	241.2	***	108.0	***	141.5	***	538	***	275	***	273	***
*Log likelihood*	-24630		-24779		-24736		-24601		-24854		-24811	

Note: The stars in the table (***, **, *) show significance at the 1%, 5% and 10% levels and standard errors are presented in parentheses.

**Table 2:** Impact of government funding on 8-year patent renewal decisions (PatentRenew8) in Canada–Regression results of the ivtobit and ivprobit model.

*Variables*	ivtobit dependent variable: PatentRenew8						ivprobit dependent variable: dPatentRenew8					
	(1)		(2)		(3)		(1)		(2)		(3)	
*ln(PubFunding)*_*t-1*_	0.3090	***	0.3119	***	0.2999	***	0.1826	***	0.1837	***	0.1830	***
	(0.0490)		(0.0478)		(0.0450)		(0.0073)		(0.0072)		(0.0072)	
*ln(nbPatCum)*_*t*_	0.4111	***					0.2868	***				
	(0.0760)						(0.0562)					
*ln (AvgCitPerPat)*_*t*_			0.5781						0.4328	*		
			(0.3528)						(0.2295)			
*[ln (AvgCitPerPat)*_*t*_*]*^*2*^			-0.2494						-0.1934			
			(0.2094)						(0.1455)			
*ln (AvgClaimPerPat)*_*t*_					0.9141	***					0.6631	***
					(0.2119)						(0.1674)	
*[ln (AvgClaimPerPat)*_*t*_*]*^*2*^					-0.5164	***					-0.3624	***
					(0.1148)						(0.0916)	
*dQC*	0.8349	***	0.8512	***	0.8057	***	0.4898	***	0.4988	***	0.4883	***
	(0.1994)		(0.1998)		(0.1892)		(0.0864)		(0.0876)		(0.0876)	
*dON*	0.9500	***	0.9384	***	0.9046	***	0.5619	***	0.5503	***	0.5497	***
	(0.1938)		(0.1939)		(0.1842)		(0.0766)		(0.0793)		(0.0791)	
*dBC*	1.0938	***	1.1631	***	1.1091	***	0.6647	***	0.7058	***	0.6975	***
	(0.2391)		(0.2435)		(0.2308)		(0.1110)		(0.1202)		(0.1180)	
*dAL*	1.1416	***	1.2465	***	1.1754	***	0.7000	***	0.7614	***	0.7445	***
	(0.3397)		(0.3538)		(0.3370)		-3.2759	***	-2.9230	***	-2.9236	***
*Constant*	-5.4616	***	-4.9624	***	-4.7919	***	-3.2759	***	-2.9230	***	-2.9236	***
	(0.6321)		(0.6053)		(0.5726)		(0.1152)		(0.0834)		(0.0832)	
***First stage***: ***ln(PubFunding)***_***t-1***_												
*ln(nbPatCum)*_*t*_	-0.3205						-0.3215					
	(0.2233)						(0.2233)					
*ln (AvgCitPerPat)*_*t*_			-0.4912						-0.4937			
			(0.7833)						(0.7837)			
*[ln (AvgCitPerPat)*_*t*_*]*^*2*^			0.0219						0.0222			
			(0.3670)						(0.3671)			
*ln (AvgClaimPerPat)*_*t*_					-0.3809						-0.3837	
					(0.2424)						(0.2427)	
*[ln (AvgClaimPerPat)*_*t*_*]*^*2*^					0.2076	***					0.2080	***
					(0.0721)						(0.0722)	
*dQC*	-2.1940	***	-2.2279	***	-2.2004	***	-2.1925	***	-2.2318	***	-2.2031	***
	(0.3637)		(0.3652)		(0.3647)		(0.3639)		(0.3657)		(0.3652)	
*dON*	-2.2895	***	-2.3150	***	-2.3033	***	-2.2887	***	-2.3199	***	-2.3067	***
	(0.3522)		(0.3539)		(0.3538)		(0.3526)		(0.3545)		(0.3544)	
*dBC*	-2.4726	***	-2.5291	***	-2.5185	***	-2.4717	***	-2.5349	***	-2.5225	***
	(0.5057)		(0.5038)		(0.5027)		(0.5058)		(0.5041)		(0.5030)	
*dAL*	-3.1668	***	-3.2118	***	-3.1792	***	-3.1657	***	-3.2170	***	-3.1828	***
	(0.7300)		(0.7228)		(0.7205)		(0.7303)		(0.7231)		(0.7209)	
*dCAResearchChair*_*t*_	1.7823	***	1.6313	***	1.7032	***	1.7837	***	1.6233	***	1.6946	***
	(0.4483)		(0.4496)		(0.4500)		(0.4549)		(0.4568)		(0.4562)	
*ResearchCareerAge*_*t*_	0.8796	***	0.8569	***	0.9052	***	0.8747	***	0.8495	***	0.8981	***
	(0.0570)		(0.0565)		(0.0594)		(0.0576)		(0.0572)		(0.0602)	
*[ResearchCarerAge*_*t*_*]*^*2*^	-0.0260	***	-0.0252	***	-0.0270	***	-0.0258	***	-0.0248	***	-0.0266	***
	(0.0023)		(0.0023)		(0.0024)		(0.0024)		(0.0023)		(0.0024)	
*ln(nbArtCum*_*t*_*)*	-0.8866	***	-0.8205	***	-0.8607	***	-0.8890	***	-0.8110	***	-0.8525	***
	(0.2286)		(0.2321)		(0.2360)		(0.2316)		(0.2371)		(0.2404)	
*[ln(nbArtCum*_*t*_*)]*^*2*^	0.1700	***	0.1604	***	0.1676	***	0.1719	***	0.1609	***	0.1678	***
	(0.0567)		(0.0570)		(0.0580)		(0.0577)		(0.0584)		(0.0593)	
*Constant*	5.5803	***	5.3559	***	5.0526	***	5.5892	***	5.3686	***	5.0659	***
	(0.4749)		(0.4637)		(0.4781)		(0.4748)		(0.4638)		(0.4784)	
*ln(α)*	-0.3148	***	-0.3166	***	-0.3039	***						
	(0.0494)		(0.0478)		(0.0451)							
*Nb observations*	7664		7664		7664		7664		7664		7664	
*Wald* χ^*2*^	90.2	***	77.0	***	93.1	***	1211	***	1227	***	1285	***
*Log likelihood*	-23606		-23649		-23621		-23604		-23668		-23639	

Note: The stars in the table (***, **, *) show significance at the 1%, 5% and 10% levels and standard errors are presented in parentheses.

**Table 3:** Impact of government funding on 12-year patent renewal decisions (PatentRenew12) in Canada–Regression results of the ivtobit and probit model.

*Variables*	ivtobit dependent variable: PatentRenew12						ivprobit dependent variable: dPatentRenew12					
	(1)		(2)		(3)		(1)		(2)		(3)	
*ln(PubFunding)*_*t-1*_	0.5534	***	0.5084	***	0.5001	***	0.1905	***	0.1906	***	0.1906	***
	(0.1668)		(0.1442)		(0.1399)		(0.0079)		(0.0082)		(0.0082)	
*ln(nbPatCum)*_*t*_	0.5062	***					0.1810	***				
	(0.1418)						(0.0598)					
*ln (AvgCitPerPat)*_*t*_			7.7179	***					3.0412	***		
			(2.1458)						(1.1290)			
*[ln (AvgCitPerPat)*_*t*_*]*^*2*^			-15.9362	***					-6.2562	**		
			(5.4814)						(2.7264)			
*ln (AvgClaimPerPat)*_*t*_					0.8382	**					0.3661	**
					(0.4079)						(0.1783)	
*[ln (AvgClaimPerPat)*_*t*_*]*^*2*^					-0.4235	**					-0.1748	*
					(0.2070)						(0.0899)	
*dQC*	1.5654	***	1.4628	***	1.4291	***	0.4847	***	0.4953	***	0.4917	***
	(0.5155)		(0.4658)		(0.4491)		(0.0914)		(0.0927)		(0.0924)	
*dON*	1.6770	***	1.5363	***	1.5173	***	0.5237	***	0.5229	***	0.5254	***
	(0.5444)		(0.4827)		(0.4673)		(0.0883)		(0.0910)		(0.0904)	
*dBC*	2.0439	***	2.0017	***	1.9647	***	0.6704	***	0.7198	***	0.7162	***
	(0.6106)		(0.5505)		(0.5347)		(0.1231)		(0.1352)		(0.1346)	
*dAL*	2.3061	***	2.2155	***	2.1701	***	0.6704	***	0.7198	***	0.7162	***
	(0.8044)		(0.7001)		(0.6839)		(0.1231)		(0.1352)		(0.1346)	
*Constant*	-9.5947	***	-8.3864	***	-8.2561	***	-3.2538	***	-3.0918	***	-3.0896	***
	(2.1487)		(1.8372)		(1.7807)		(0.2187)		(0.1676)		(0.1673)	
***First stage***: ***ln(PubFunding)***_***t-1***_												
*ln(nbPatCum)*_*t*_	-0.3151						-0.2898					
	(0.2225)						(0.2299)					
*ln (AvgCitPerPat)*_*t*_			-0.4136						-0.2111			
			(0.7806)						(0.8811)			
*[ln (AvgCitPerPat)*_*t*_*]*^*2*^			-0.0102						-0.1785			
			(0.3668)						(0.4422)			
*ln (AvgClaimPerPat)*_*t*_					-0.3649						-0.4891	*
					(0.2408)						(0.2537)	
*[ln (AvgClaimPerPat)*_*t*_*]*^*2*^					0.1992	***					0.2249	***
					(0.0717)						(0.0763)	
*dQC*	-2.1363	***	-2.1476	***	-2.1241	***	-1.9135	***	-1.9288	***	-1.9086	***
	(0.3639)		(0.3653)		(0.3649)		(0.3793)		(0.3809)		(0.3811)	
*dON*	-2.2284	***	-2.2292	***	-2.2214	***	-1.9897	***	-1.9953	***	-1.9956	***
	(0.3536)		(0.3561)		(0.3558)		(0.3693)		(0.3722)		(0.3719)	
*dBC*	-2.4032	***	-2.4302	***	-2.4245	***	-2.2338	***	-2.2652	***	-2.2620	***
	(0.5060)		(0.5046)		(0.5032)		(0.5430)		(0.5413)		(0.5404)	
*dAL*	-3.1062	***	-3.1238	***	-3.0960	***	-2.9587	***	-2.9784	***	-2.9505	***
	(0.7317)		(0.7251)		(0.7230)		(0.7764)		(0.7706)		(0.7708)	
*dCAResearchChair*_*t*_	1.7476	***	1.7669	***	1.7868	***	1.6219	***	1.6370	***	1.6482	***
	(0.5553)		(0.5471)		(0.5443)		(0.5777)		(0.5650)		(0.5665)	
*ResearchCareerAge*_*t*_	0.8259	***	0.8074	***	0.8557	***	0.8263	***	0.8060	***	0.8435	***
	(0.0578)		(0.0581)		(0.0608)		(0.0616)		(0.0620)		(0.0646)	
*[ResearchCarerAge*_*t*_*]*^*2*^	-0.0230	***	-0.0224	***	-0.0242	***	-0.0230	***	-0.0223	***	-0.0237	***
	(0.0023)		(0.0024)		(0.0025)		(0.0025)		(0.0025)		(0.0026)	
*ln(nbArtCum*_*t*_*)*	-0.9450	***	-0.9293	***	-0.9622	***	-0.8069	***	-0.7799	***	-0.7994	***
	(0.2467)		(0.2525)		(0.2551)		(0.2438)		(0.2522)		(0.2541)	
*[ln(nbArtCum*_*t*_*)]*^*2*^	0.1807	***	0.1775	***	0.1837	***	0.1500	**	0.1449	**	0.1486	**
	(0.0607)		(0.0630)		(0.0633)		(0.0594)		(0.0625)		(0.0624)	
*Constant*	5.6830	***	5.4573	***	5.1608	***	5.4524	***	5.2562	***	5.0223	***
	(0.4733)		(0.4598)		(0.4749)		(0.4989)		(0.4842)		(0.5010)	
*ln(α)*	-0.5710	***	-0.5198	***	-0.5113	***						
	(0.1704)		(0.1469)		(0.1427)							
*Nb observations*	7664		7664		7664		7664		7664		7664	
*Wald χ*^*2*^	27.4	***	41.4	***	187.6	***	1197	***	1830	***	1289	***
*Log likelihood*	-23064		-23075		-23064		-21185		-21197		-21191	

Note: The stars in the table (***, **, *) show significance at the 1%, 5% and 10% levels and standard errors are presented in parentheses.

We formulated the expectation that highly cited patents are correlated with highly valued patents. Our results in Table 1, Table 2 and Table 3 confirm these expectations. A higher number of citations eventually yields a patent that is more likely to be commercialized in the market. For that reason, these patents are more likely to be renewed after 4 years. In regard to the number of citations, at first sight, we found that receiving more citations positively influences the patent renewal decisions after 4 years (Model 2 in Table 1), 8 years (Model 2 in Table 2) and even 12 years (Model 2 in Table 3). We investigated whether non-linear effects would better fit our model. Only in the regressions for the final renewal stage is the quadratic term significant and despite its negative sign, the overall effect remains positive except for the renewal in 12 years (exhibiting diminishing returns). For the first and second renewal stages (after 4 and 8 years), we found a linear and positive impact of the number of citations which suggests that patent renewal decisions benefit from a greater number of patent citations, i.e. patents of a greater quality are more likely to be renewed, hence providing support for our first hypothesis.

The third columns of each table also show that the number of claims positively increase the probability of renewals but when we include non-linear effects, the results are mostly located along the decreasing part of an inverted U-shaped curve. Regarding this non-linear effect, it is important to note that if the patents of an academic inventor have more claims (more than 3 claims in our sample), they are not more likely to be renewed in subsequent years. These findings provide partial support for our second hypothesis. This finding is likely to be linked to the fact that fees vary for excessive claims. There is a large additional fee if a patent documents make multiple claims, and in this case the fees include an additional cost per extra claim in excess of 20 claims in the USPTO (the excess claims fees are charged in excess of three for each independent claim). The negative effect is likely due to these excess claims.

Our third hypothesis examined the influence of public funding. Not surprisingly, our findings support the fact that public funding is associated with a positive impact on patent renewals. In other words, when academic inventors raise more public funding, they have more patents that are renewed in subsequent years. We found significant results for all 4-year, 8-year and 12-year renewal decisions. This could be due to the fact that government funding has a positive effect on patent quality [53]. Higher value patents are more likely to be extended as long as they give benefits to the owner. Public funding has a direct effect on patent quality, and these patents have a higher chance to be renewed. These results validate our third hypothesis and clearly demonstrate the positive correlation between public funding and patent renewal decisions.

Turning now briefly to our control variables, we find that the chances of renewing a patent are higher when academic inventors have more patents, i.e. there is a positive correlation between the total number of patents (*nbPatCum*) and patent renewals in all three tables (Tables 1, 2 and 3).

Universities are significant bases for the development of technological innovation and enhancing their invention capacity is an important development challenge. Facing these renewal decisions, one can raise the question as to where funding yields the highest impact on renewal decisions in universities in all provinces of Canada. Our results show that the four provinces of Quebec (*dQC*), Ontario (*dON*), British Columbia (*dBC*) and Alberta (*dAL*) are significantly different from the other provinces (*dCanada_others* is the omitted dummy variable). Thus inventors from the universities of these provinces have higher renewal rates compared to the other provinces.

Our last model, takes the analysis at the patent level (rather than at the academic inventor level). Table 4 shows the results of Cox proportional hazard regressions. We report three models including control variables and year dummies. In all three models, the hazard ratio is greater than one and the coefficient of average funding is significant and positive. In addition, average funding that was allocated to patents increase the likelihood of renewals. These findings are consistent with our previous funding at the individual level (please note that we apply this survival analysis only for robustness check because there are some issues when patents are analyzed individually in terms of funding. Funding generally comes to researchers and not specific patents and for academic researchers, this funding goes to both publications and patents and allocating funding to each of them is the main issue).

**Table 4:** Impact of government funding on patent renewal decisions–results of Cox proportional hazards.

*Variables*	*Model (1)*			*Model (2)*			*Model (3)*		
	*Parameter Estimate*		*Hazard Ratio (HR)* *[95% CI for HR]*	*Parameter Estimate*		*Hazard Ratio (HR)* *[95% CI for HR]*	*Parameter Estimate*		*Hazard Ratio (HR)* *[95% CI for HR]*
*ln(AvgPubFund)*	0.0056	*	1.0055	0.0056	*	1.0056	0.0054	*	1.0054
	(0.0031)		[0.9995, 1.0117]	(0.0031)		[0.9995, 1.0118]	(0.0031)		[0.9992, 1.0115]
*ln (NbAssignees)*	-0.1302		0.8779	-0.1200		0.8869	-0.1278		0.8800
	(0.0863)		[0.7413, 1.0397]	(0.0866)		[0.7484, 1.0511]	(0.0863)		[0.7431, 1.0423]
*ln (NbInventors)*	-0.1032	**	0.9020	-0.1046	**	0.9007	-0.1008	**	0.9041
	(0.0428)		[0.8293, 0.9810]	(0.0429)		[0.8280, 0.9797]	(0.0430)		[0.8310, 0.9836]
*ln(ForwardCit)*	-0.1540	***	0.8573				-0.1519	***	0.8591
	(0.0198)		[0.8246, 0.8912]				(0.0201)		[0.8260, 0.8936]
*ln (BackwardCit)*	-0.0086		0.9914				-0.0080		0.9920
	(0.0233)		[0.9472, 1.0377]				(0.0233)		[0.9477, 1.0384]
*ln (NbClaims)*				-0.0505	*	0.9507	-0.0171		0.9831
				(0.0259)		[0.9036, 1.0003]	(0.0264)		]0.9334, 1.0353]
*Year dummies*	Yes			Yes			Yes		
*Nb observations*	3659			3659			3569		
*Wald χ*^*2*^	746	***		688	***		746	***	
*Log likelihood*	-23414			-23443			-23441		

Note: The stars in the table (***, **, *) show significance at the 1%, 5% and 10% levels and standard errors are presented in parentheses.

**Fig 3:**
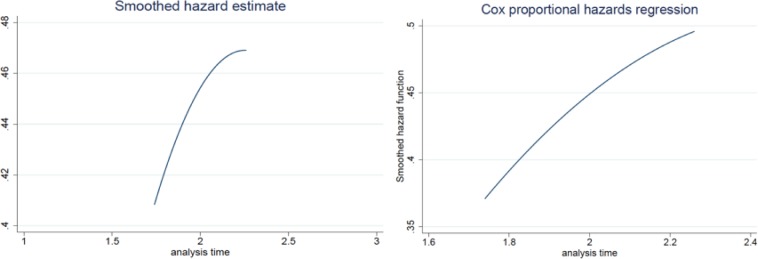
**Hazard function curve (a: smoothed hazard estimate and b: cox proportional hazards regression), analysis time indicates the renewal decisions at time *t*, (*t* equals 1 for the renewal after 4 years, *t* equals 2 for the renewal after 8 years and equals 3 for the renewal after 12 years)**.

Fig 3 illustrates the smoothed hazard function and smoothed hazard estimates of our model estimated using a kernel method. This actually plots the log estimated hazards and the curve uses the mean values of predictors. The plot suggests that the risk of death for patents as the hazard increases over time. The analysis time indicates the renewal decisions at time *t* where *t* equals 1 for the renewal after 4 years, *t* equals 2 for the renewal after 8 years and equals 3 for the renewal after 12 years.

## Discussion and conclusion

This empirical study explores the question of the impact of public funding on patent renewals over their lifetime. For this purpose, we employed a sample of Canadian nanotechnology patents. Our study compiled the academic patents to which Canadian academic inventors have contributed. Our dataset enabled us to extend our research in various directions: first, to analyze whether the number of citations received by patents is a valid indicator of economic value of inventions; second, to test the impact of the number of claims on patent quality; third, to empirically investigate whether public policies and funding programs affect patent values using the renewal of patents.

We indeed find that government-funded research in universities is an important consideration when we examine academic patents. With this data, we have shown that the amount of funding received by an academic inventor exhibits a positive effect on the tendency to renew their patents. The effect of funding programs on the value of granted patents is to enhance the probability of renewing patents in subsequent years and encourage inventors with great commercialization opportunities. In universities, because government accounts for a comprehensively larger amount of funding, it is important to disentangle the role of government in the economic value of patents; this is undoubtedly the most important finding of this paper. By matching renewal information data with bibliometric analysis of patent statistics, we are able to evaluate the policy impacts on the quality and value of patents. To the best of our knowledge, no such analysis has been performed on the relationship between funding and renewal decisions. This gives a clear view on how public funding serves as an indicator of invention output of academic research, and it highlights to what extent government incentives can be fruitful for researchers. Our detailed examination reveals insights on effective policy regarding the market value of patents. University patents are usually slow to acknowledge the effort required to transform a patent into a commercial product. More substantial work is necessary to increase the opportunities for a patent to be commercialized in industry. Applicability and marketability of a granted patent is a time related process, and inventors need to maintain their inventions for a longer time before they are commercially viable. Due to the fact that university funds mainly come from government, government should not only intensify the funding of inventive activities, but also make the relevant supporting policies to provide a long-term funding mechanism for the innovation of academic inventors.

In regard to number of claims, our results provide useful policy designs to encourage strategies to narrow patent claims. This study shows that perceived importance of the claims is not associated with patent value in terms of renewal decisions. Interestingly, regarding the challenge of assessing the effect of the number of claims in determining patent value, we cannot find a positive impact of claims on patent renewal decisions in subsequent years after the grant year despite the relationship of number of claims to patent quality. Most interestingly, our model explains patent citations as an indicator of patent value with a strong explanatory power for patent renewals.

However, the limitations of our study cannot be neglected as they affect the generalization of our findings to all university patents. First, our sample data was drawn from a population of nanotechnology university patents, and generalization to other fields should be done cautiously. Second, although patent renewals are good indicators of patent value, they are not perfect. Inventors can maintain their patents for a longer time even if they have low value, but this is difficult to identify. Finally, one needs to verify whether private funding has an effect on these renewal decisions. Our data suffers from a lack of information regarding private funding, as the private sector might be more interested than the public sector in technological inventions from universities. And often, private funding represents direct commands for the solving of specific industrial problems. Lacking this data, we are unable to say how such an effect would influence our findings.

Although this study provides insights about funding and patent value in universities, a number of other avenues for future research are open. One would have to rule out the other factors that play a role in patent renewal decisions in universities. One such factor is the market potential of these inventions. Market maturity is expected to be an important indicator in renewal decisions. One would expect that the potential market of the granted patent is at early stages and it is likely to have a successful market for a long time or on the opposite, it could be in the late stage and may have less success in the market. There is also a need to find out how government funding acts in reducing researchers’ financial constraints to pay the corresponding renewal fees. This would require funding information for the entire duration of the period, i.e. from 1995 to 2017.

## Supporting information

S1 Table Correlation matrix. (DOCX)

S2 Table Descriptive statistics. (DOCX)

S3 Table Impact of government funding on 4-year patent renewal decisions (*PatentRenew4*) in Canada–Regression results of the OLS model (dependent variable: PatentRenew4). (DOCX)

S4 Table Impact of government funding on 8-year patent renewal decisions (*PatentRenew8*) in Canada–Regression results of the OLS model. (DOCX)

S5 Table Impact of government funding on 12-year patent renewal decisions (*PatentRenew12*) in Canada–Regression results of the ivtobit model. (DOCX)

S6 Table Impact of government funding on 4-year patent renewal decisions (*NumPatentRenew4*) in Canada–Regression results of the ivtobit and ivprobit model. (DOCX)

S7 Table Impact of government funding on 8-year patent renewal decisions (*NumPatentRenew8*) in Canada–Regression results of the ivtobit and ivprobit model. (DOCX)

S8 Table Impact of government funding on 12-year patent renewal decisions (*NumPatentRenew12*) in Canada–Regression results of the ivtobit and probit model. (DOCX)
