# Supplementary material for: Do patents of academic funded researchers enjoy a longer life? A study of patent renewal decisions
Source: PLoS One. 2018 Aug 29;13(8):e0202643. doi: 10.1371/journal.pone.0202643 (PMC6114791; doi:10.1371/journal.pone.0202643)
Supplement: S1 Table — (DOCX) [file pone.0202643.s001.docx]

**S1 Table. Correlation matrix**

|  |  | 1 | 2 | 3 | 4 | 5 | 6 | 7 | 8 | 9 | 10 |
| --- | --- | --- | --- | --- | --- | --- | --- | --- | --- | --- | --- |
| *PatentRenew4* | 1 | 1 |  |  |  |  |  |  |  |  |  |
| *PatentRenew8* | 2 | -0.0143 | 1 |  |  |  |  |  |  |  |  |
| *PatentRenew12* | 3 | -0.0105 | -0.0062 | 1 |  |  |  |  |  |  |  |
| *ln(PubFunding)* | 4 | 0.0454 | 0.0422 | 0.0067 | 1 |  |  |  |  |  |  |
| *ln (AvgCitPerPat)* | 5 | -0.0196 | -0.0169 | -0.0103 | -0.058 | 1 |  |  |  |  |  |
| *ln (AvgClaimPerPat)* | 6 | -0.0866 | -0.057 | -0.0262 | -0.1301 | 0.2216 | 1 |  |  |  |  |
| *ln(nbPatCum)* | 7 | 0.0612 | 0.0195 | -0.006 | 0.0418 | 0.2331 | 0.0569 | 1 |  |  |  |
| *ResearchCareerAge* | 8 | 0.0583 | 0.1047 | 0.079 | 0.4074 | -0.0454 | -0.2434 | 0.1822 | 1 |  |  |
| *CAResearchChair* | 9 | 0.0091 | -0.0031 | -0.0084 | 0.1164 | -0.0042 | -0.0088 | -0.0052 | 0.0251 | 1 |  |
| *ln(nbArtCum)* | 10 | 0.0327 | 0.0002 | -0.0238 | 0.0185 | 0.0381 | -0.016 | 0.1572 | 0.2251 | 0.0925 | 1 |
