# Supplementary material for: Do patents of academic funded researchers enjoy a longer life? A study of patent renewal decisions
Source: PLoS One. 2018 Aug 29;13(8):e0202643. doi: 10.1371/journal.pone.0202643 (PMC6114791; doi:10.1371/journal.pone.0202643)
Supplement: S2 Table — (DOCX) [file pone.0202643.s002.docx]

**S2 Table. Descriptive statistics**

|  | Count | Mean | Std. Dev. | Min | Max |
| --- | --- | --- | --- | --- | --- |
| *PatentRenew4* | 7,664 | 0.0347 | 0.1523 | 0 | 2 |
| *PatentRenew8* | 7,664 | 0.0127 | 0.0964 | 0 | 1 |
| *PatentRenew12* | 7,664 | 0.0035 | 0.0543 | 0 | 1 |
| *PubFunding* | 7,664 | 229,598 | 879,720 | 0 | 22,400,000 |
| *AvgCitPerPat* | 7,664 | 0.0831 | 0.7250 | 0 | 21 |
| *AvgClaimPerPat* | 7,664 | 2.9906 | 8.9535 | 0 | 145 |
| *nbPatCum* | 7,664 | 2.6850 | 4.9760 | 1 | 62 |
| *ResearchCareerAge* | 7,664 | 9.5427 | 5.3513 | 1 | 22 |
| *CAResearchChair* | 7,664 | 0.0282 | 0.2250 | 0 | 2 |
| *nbArtCum* | 7,664 | 4.5920 | 13.8266 | 0 | 260 |
