# Supplementary material for: Do patents of academic funded researchers enjoy a longer life? A study of patent renewal decisions
Source: PLoS One. 2018 Aug 29;13(8):e0202643. doi: 10.1371/journal.pone.0202643 (PMC6114791; doi:10.1371/journal.pone.0202643)
Supplement: S3 Table — (DOCX) [file pone.0202643.s003.docx]

S3 Table. Impact of government funding on 4-year patent renewal decisions (*PatentRenew4*) in Canada – Regression results of the OLS model (dependent variable : PatentRenew4)

| ***Variables*** | **Ordinary least squares (OLS)**  **reg** | | | | | |  | **Two-stage least squares (2SLS)**  **ivregress** | | | | | |
| --- | --- | --- | --- | --- | --- | --- | --- | --- | --- | --- | --- | --- | --- |
|  | **(1)** | | **(2)** | | **(3)** | |  | **(1)** | | **(2)** | | **(3)** | |
| *ln(PubFunding)_t-1_* | 0.0002 |  | 0.0001 |  | 0.0002 |  |  | 0.0045 | *** | 0.0051 | *** | 0.0040 | *** |
|  | (0.0003) |  | (0.0003) |  | (0.0003) |  |  | (0.0006) |  | (0.0006) |  | (0.0006) |  |
| *ln(nbPatCum)_t_* | 0.0094 | *** |  |  |  |  |  | 0.0109 | *** |  |  |  |  |
|  | (0.0019) |  |  |  |  |  |  | (0.0021) |  |  |  |  |  |
| *ln (AvgCitPerPat)_t_* |  |  | -0.0269 | *** |  |  |  |  |  | -0.0216 | *** |  |  |
|  |  |  | (0.0060) |  |  |  |  |  |  | (0.0072) |  |  |  |
| *[ln (AvgCitPerPat)_t_]^2^* |  |  | 0.0115 | ** |  |  |  |  |  | 0.0101 | ** |  |  |
|  |  |  | (0.0045) |  |  |  |  |  |  | (0.0048) |  |  |  |
| *ln (AvgClaimPerPat)_t_* |  |  |  |  | -0.0108 | *** |  |  |  |  |  | -0.0089 | *** |
|  |  |  |  |  | (0.0024) |  |  |  |  |  |  | (0.0027) |  |
| *[ln (AvgClaimPerPat)_t_]^2^* |  |  |  |  | 0.0017 | *** |  |  |  |  |  | 0.0004 |  |
|  |  |  |  |  | (0.0006) |  |  |  |  |  |  | (0.0007) |  |
| *dQC* | 0.0046 |  | 0.0042 |  | 0.0037 |  |  | 0.0160 | *** | 0.0178 | *** | 0.0147 | *** |
|  | (0.0031) |  | (0.0031) |  | (0.0031) |  |  | (0.0040) |  | (0.0041) |  | (0.0038) |  |
| *dON* | 0.0043 |  | 0.0036 |  | 0.0030 |  |  | 0.0171 | *** | 0.0186 | *** | 0.0155 | *** |
|  | (0.0031) |  | (0.0032) |  | (0.0032) |  |  | (0.0039) |  | (0.0041) |  | (0.0038) |  |
| *dBC* | -0.0039 |  | -0.0039 |  | -0.0042 |  |  | 0.0101 | ** | 0.0126 | ** | 0.0096 | * |
|  | (0.0041) |  | (0.0042) |  | (0.0042) |  |  | (0.0051) |  | (0.0052) |  | (0.0050) |  |
| *dAL* | -0.0012 |  | -0.0012 |  | -0.0015 |  |  | 0.0146 | ** | 0.0174 | ** | 0.0135 | ** |
|  | (0.0058) |  | (0.0059) |  | (0.0058) |  |  | (0.0071) |  | (0.0073) |  | (0.0067) |  |
| *dCAResearchChair_t_* | 0.0055 |  | 0.0048 |  | 0.0049 |  |  |  |  |  |  |  |  |
|  | (0.0135) |  | (0.0135) |  | (0.0135) |  |  |  |  |  |  |  |  |
| *ResearchCareerAge_t_* | 0.0083 | *** | 0.0087 | *** | 0.0075 | *** |  |  |  |  |  |  |  |
|  | (0.0008) |  | (0.0008) |  | (0.0008) |  |  |  |  |  |  |  |  |
| *[ResearchCarerAge_t_]^2^* | -0.0004 | *** | -0.0004 | *** | -0.0003 | *** |  |  |  |  |  |  |  |
|  | (0.0000) |  | (0.0000) |  | (0.0000) |  |  |  |  |  |  |  |  |
| *ln(nbArtCum_t_)* | -0.0087 | *** | -0.0078 | *** | -0.0070 | ** |  |  |  |  |  |  |  |
|  | (0.0028) |  | (0.0029) |  | (0.0028) |  |  |  |  |  |  |  |  |
| *[ln(nbArtCum_t_)]^2^* | 0.0025 | *** | 0.0025 | *** | 0.0024 | *** |  |  |  |  |  |  |  |
|  | (0.0008) |  | (0.0008) |  | (0.0008) |  |  |  |  |  |  |  |  |
| *Constant* | -0.0182 | *** | -0.0110 | ** | -0.0042 |  |  | -0.0339 | *** | -0.0288 | *** | -0.0157 | ** |
|  | (0.0052) |  | (0.0051) |  | (0.0052) |  |  | (0.0077) |  | (0.0080) |  | (0.0076) |  |
| *Nb observations* | 7664 |  | 7664 |  | 7664 |  |  | 7664 |  | 7664 |  | 7664 |  |
| *Wald χ^2^* |  |  |  |  |  |  |  | 151 |  | 126 |  | 307 |  |
| *Log likelihood* | 6068 |  | 6061 |  | 6070 |  |  | -24601 |  | -24854 |  | -24811 |  |

Notes: ***, **, * show significance at the 1%, 5% and 10% levels and standard errors are presented in parentheses. We use *dCAResearchChair, ResearchCareerAge* and *ln(nbArtCum)* as instrument variables in ivregress model.
