# Supplementary material for: Do patents of academic funded researchers enjoy a longer life? A study of patent renewal decisions
Source: PLoS One. 2018 Aug 29;13(8):e0202643. doi: 10.1371/journal.pone.0202643 (PMC6114791; doi:10.1371/journal.pone.0202643)
Supplement: S4 Table — (DOCX) [file pone.0202643.s004.docx]

S4 Table. Impact of government funding on 8-year patent renewal decisions (*PatentRenew8*) in Canada – Regression results of the OLS model

| ***Variables*** | **Ordinary least squares (OLS)**  **reg** | | | | | |  | **Two-stage least squares (2SLS)**  **ivregress** | | | | | |
| --- | --- | --- | --- | --- | --- | --- | --- | --- | --- | --- | --- | --- | --- |
|  | **(1)** | | **(2)** | | **(3)** | |  | **(1)** | | **(2)** | | **(3)** | |
| *ln(PubFunding)_t-1_* | -0.0002 |  | -0.0002 |  | -0.0002 |  |  | 0.0044 | *** | 0.0051 | *** | 0.0041 | *** |
|  | (0.0002) |  | (0.0002) |  | (0.0002) |  |  | (0.0005) |  | (0.0006) |  | (0.0005) |  |
| *ln(nbPatCum)_t_* | 0.0002 |  |  |  |  |  |  | 0.0014 |  |  |  |  |  |
|  | (0.0012) |  |  |  |  |  |  | (0.0015) |  |  |  |  |  |
| *ln (AvgCitPerPat)_t_* |  |  | -0.0088 | *** |  |  |  |  |  | -0.0216 | *** |  |  |
|  |  |  | (0.0033) |  |  |  |  |  |  | (0.0072) |  |  |  |
| *[ln (AvgCitPerPat)_t_]^2^* |  |  | 0.0031 | ** |  |  |  |  |  | 0.0101 | ** |  |  |
|  |  |  | (0.0014) |  |  |  |  |  |  | (0.0048) |  |  |  |
| *ln (AvgClaimPerPat)_t_* |  |  |  |  | -0.0036 | *** |  |  |  |  |  | -0.0025 | * |
|  |  |  |  |  | (0.0012) |  |  |  |  |  |  | (0.0015) |  |
| *[ln (AvgClaimPerPat)_t_]^2^* |  |  |  |  | 0.0008 | ** |  |  |  |  |  | 0.0000 |  |
|  |  |  |  |  | (0.0003) |  |  |  |  |  |  | (0.0004) |  |
| *dQC* | 0.0034 | * | 0.0033 | * | 0.0032 |  |  | 0.0119 | *** | 0.0178 | *** | 0.0111 | *** |
|  | (0.0020) |  | (0.0020) |  | (0.0020) |  |  | (0.0030) |  | (0.0041) |  | (0.0028) |  |
| *dON* | 0.0048 | ** | 0.0048 | ** | 0.0046 | ** |  | 0.0138 | *** | 0.0186 | *** | 0.0130 | *** |
|  | (0.0020) |  | (0.0020) |  | (0.0020) |  |  | (0.0031) |  | (0.0041) |  | (0.0030) |  |
| *dBC* | 0.0051 | * | 0.0050 | * | 0.0049 | * |  | 0.0147 | *** | 0.0126 | ** | 0.0139 | *** |
|  | (0.0030) |  | (0.0030) |  | (0.0030) |  |  | (0.0040) |  | (0.0052) |  | (0.0038) |  |
| *dAL* | -0.0007 |  | -0.0007 |  | -0.0007 |  |  | 0.0122 | ** | 0.0174 | ** | 0.0112 | ** |
|  | (0.0029) |  | (0.0030) |  | (0.0029) |  |  | (0.0048) |  | (0.0073) |  | (0.0045) |  |
| *dCAResearchChair_t_* | -0.0002 |  | -0.0002 |  | -0.0002 |  |  |  |  |  |  |  |  |
|  | (0.0061) |  | (0.0061) |  | (0.0061) |  |  |  |  |  |  |  |  |
| *ResearchCareerAge_t_* | 0.0043 | *** | 0.0044 | *** | 0.0041 | *** |  |  |  |  |  |  |  |
|  | (0.0007) |  | (0.0007) |  | (0.0007) |  |  |  |  |  |  |  |  |
| *[ResearchCarerAge_t_]^2^* | -0.0001 | *** | -0.0001 | *** | -0.0001 | *** |  |  |  |  |  |  |  |
|  | (0.0000) |  | (0.0000) |  | (0.0000) |  |  |  |  |  |  |  |  |
| *ln(nbArtCum_t_)* | -0.0066 | *** | -0.0065 | *** | -0.0063 | *** |  |  |  |  |  |  |  |
|  | (0.0021) |  | (0.0021) |  | (0.0021) |  |  |  |  |  |  |  |  |
| *[ln(nbArtCum_t_)]^2^* | 0.0011 | * | 0.0011 | * | 0.0011 | * |  |  |  |  |  |  |  |
|  | (0.0006) |  | (0.0006) |  | (0.0006) |  |  |  |  |  |  |  |  |
| *Constant* | -0.0101 | *** | -0.0099 | *** | -0.0084 | ** |  | -0.0345 | *** | -0.0288 | *** | -0.0294 | *** |
|  | (0.0034) |  | (0.0034) |  | (0.0034) |  |  | (0.0062) |  | (0.0080) |  | (0.0058) |  |
| *Nb observations* | 7664 |  | 7664 |  | 7664 |  |  | 7664 |  | 7664 |  | 7664 |  |
| *Wald χ^2^* |  |  |  |  |  |  |  | 122.6 |  | 125.9 |  | 132.0 |  |
| *Log likelihood* | 9616 |  | 9616 |  | 9618 |  |  |  |  |  |  |  |  |

Notes: ***, **, * show significance at the 1%, 5% and 10% levels and standard errors are presented in parentheses. We use *dCAResearchChair, ResearchCareerAge* and *ln(nbArtCum)* as instrument variables in ivregress model.
