# Supplementary material for: Do patents of academic funded researchers enjoy a longer life? A study of patent renewal decisions
Source: PLoS One. 2018 Aug 29;13(8):e0202643. doi: 10.1371/journal.pone.0202643 (PMC6114791; doi:10.1371/journal.pone.0202643)
Supplement: S5 Table — (DOCX) [file pone.0202643.s005.docx]

S5 Table. Impact of government funding on 12-year patent renewal decisions (*PatentRenew12*) in Canada – Regression results of the ivtobit model

| ***Variables*** | **Ordinary least squares (OLS)**  **reg** | | | | | |  | **Two-stage least squares (2SLS)**  **ivregress** | | | | | |
| --- | --- | --- | --- | --- | --- | --- | --- | --- | --- | --- | --- | --- | --- |
|  | **(1)** | | **(2)** | | **(3)** | |  | **(1)** | | **(2)** | | **(3)** | |
| *ln(PubFunding)_t-1_* | -0.0002 | ** | -0.0002 | ** | -0.0002 | ** |  | 0.0017 | *** | 0.0016 | *** | 0.0016 | *** |
|  | (0.0001) |  | (0.0001) |  | (0.0001) |  |  | (0.0004) |  | (0.0003) |  | (0.0003) |  |
| *ln(nbPatCum)_t_* | -0.0011 | * |  |  |  |  |  | -0.0008 |  |  |  |  |  |
|  | (0.0006) |  |  |  |  |  |  | (0.0007) |  |  |  |  |  |
| *ln (AvgCitPerPat)_t_* |  |  | -0.0027 | ** |  |  |  |  |  | -0.0036 | ** |  |  |
|  |  |  | (0.0012) |  |  |  |  |  |  | (0.0018) |  |  |  |
| *[ln (AvgCitPerPat)_t_]^2^* |  |  | 0.0010 | ** |  |  |  |  |  | 0.0017 | ** |  |  |
|  |  |  | (0.0005) |  |  |  |  |  |  | (0.0008) |  |  |  |
| *ln (AvgClaimPerPat)_t_* |  |  |  |  | -0.0008 |  |  |  |  |  |  | -0.0007 |  |
|  |  |  |  |  | (0.0006) |  |  |  |  |  |  | (0.0007) |  |
| *[ln (AvgClaimPerPat)_t_]^2^* |  |  |  |  | 0.0002 |  |  |  |  |  |  | 0.0001 |  |
|  |  |  |  |  | (0.0001) |  |  |  |  |  |  | (0.0002) |  |
| *dQC* | 0.0030 | ** | 0.0030 | ** | 0.0030 | ** |  | 0.0052 | *** | 0.0052 | *** | 0.0049 | *** |
|  | (0.0012) |  | (0.0012) |  | (0.0012) |  |  | (0.0017) |  | (0.0017) |  | (0.0017) |  |
| *dON* | 0.0032 | *** | 0.0032 | *** | 0.0032 | *** |  | 0.0055 | *** | 0.0055 | *** | 0.0052 | *** |
|  | (0.0012) |  | (0.0012) |  | (0.0012) |  |  | (0.0018) |  | (0.0018) |  | (0.0018) |  |
| *dBC* | 0.0028 | * | 0.0027 | * | 0.0027 | * |  | 0.0052 | *** | 0.0050 | *** | 0.0048 | *** |
|  | (0.0015) |  | (0.0015) |  | (0.0015) |  |  | (0.0019) |  | (0.0019) |  | (0.0018) |  |
| *dAL* | 0.0032 |  | 0.0032 |  | 0.0032 |  |  | 0.0071 | ** | 0.0070 | ** | 0.0067 | ** |
|  | (0.0021) |  | (0.0021) |  | (0.0021) |  |  | (0.0033) |  | (0.0033) |  | (0.0032) |  |
| *dCAResearchChair_t_* | -0.0014 |  | -0.0013 |  | -0.0013 |  |  |  |  |  |  |  |  |
|  | (0.0008) |  | (0.0008) |  | (0.0008) |  |  |  |  |  |  |  |  |
| *ResearchCareerAge_t_* | 0.0009 | ** | 0.0009 | ** | 0.0009 | ** |  |  |  |  |  |  |  |
|  | (0.0004) |  | (0.0004) |  | (0.0004) |  |  |  |  |  |  |  |  |
| *[ResearchCarerAge_t_]^2^* | 0.0000 |  | 0.0000 |  | 0.0000 |  |  |  |  |  |  |  |  |
|  | (0.0000) |  | (0.0000) |  | (0.0000) |  |  |  |  |  |  |  |  |
| *ln(nbArtCum_t_)* | -0.0043 | *** | -0.0043 | *** | -0.0043 | *** |  |  |  |  |  |  |  |
|  | (0.0010) |  | (0.0011) |  | (0.0011) |  |  |  |  |  |  |  |  |
| *[ln(nbArtCum_t_)]^2^* | 0.0007 | *** | 0.0007 | *** | 0.0007 | *** |  |  |  |  |  |  |  |
|  | (0.0002) |  | (0.0002) |  | (0.0002) |  |  |  |  |  |  |  |  |
| *Constant* | -0.0023 |  | -0.0032 |  | -0.0031 |  |  | -0.0143 | *** | -0.0148 | *** | -0.0138 | *** |
|  | (0.0024) |  | (0.0024) |  | (0.0024) |  |  | (0.0039) |  | (0.0040) |  | (0.0038) |  |
| *Nb observations* | 7664 |  | 7664 |  | 7664 |  |  | 7664 |  | 7664 |  | 7664 |  |
| *Wald χ^2^* |  |  |  |  |  |  |  | 36.6 |  | 31.6 |  | 32.3 |  |
| *Log likelihood* | 14163 |  | 14162 |  | 14162 |  |  |  |  |  |  |  |  |

Notes: ***, **, * show significance at the 1%, 5% and 10% levels and standard errors are presented in parentheses. We use *dCAResearchChair, ResearchCareerAge* and *ln(nbArtCum)* as instrument variables in ivregress model.
