# Supplementary material for: Do patents of academic funded researchers enjoy a longer life? A study of patent renewal decisions
Source: PLoS One. 2018 Aug 29;13(8):e0202643. doi: 10.1371/journal.pone.0202643 (PMC6114791; doi:10.1371/journal.pone.0202643)
Supplement: S6 Table — (DOCX) [file pone.0202643.s006.docx]

**S6 Table. Impact of government funding on 4-year patent renewal decisions (*NumPatentRenew4*) in Canada – Regression results of the ivtobit and ivprobit model**

| ***Variables*** | **ivtobit**  **dependent variable:** ***NumPatentRenew4*** | | | | | |  | **ivprobit**  **dependent variable:** ***dNumPatentRenew4*** | | | | | |
| --- | --- | --- | --- | --- | --- | --- | --- | --- | --- | --- | --- | --- | --- |
|  | **(1)** | | **(2)** | | **(3)** | |  | **(1)** | | **(2)** | | **(3)** | |
| *ln(PubFunding)_t-1_* | 0.1443 | *** | 0.2486 | *** | 0.2133 | *** |  | 0.1014 | *** | 0.1294 | *** | 0.1186 | *** |
|  | (0.0233) |  | (0.0334) |  | (0.0311) |  |  | (0.0127) |  | (0.0108) |  | (0.0119) |  |
| *ln(nbPatCum)_t_* | 0.9353 | *** |  |  |  |  |  | 0.6614 | *** |  |  |  |  |
|  | (0.0521) |  |  |  |  |  |  | (0.0484) |  |  |  |  |  |
| *ln (AvgCitPerPat)_t_* |  |  | 1.2515 | ** |  |  |  |  |  | 0.6566 | ** |  |  |
|  |  |  | (0.5234) |  |  |  |  |  |  | (0.2834) |  |  |  |
| *[ln (AvgCitPerPat)_t_]^2^* |  |  | -0.5735 | * |  |  |  |  |  | -0.3101 | * |  |  |
|  |  |  | (0.3392) |  |  |  |  |  |  | (0.1840) |  |  |  |
| *ln (AvgClaimPerPat)_t_* |  |  |  |  | 1.0740 | *** |  |  |  |  |  | 0.5922 | *** |
|  |  |  |  |  | (0.2304) |  |  |  |  |  |  | (0.1302) |  |
| *[ln (AvgClaimPerPat)_t_]^2^* |  |  |  |  | -0.5473 | *** |  |  |  |  |  | -0.3043 | *** |
|  |  |  |  |  | (0.0994) |  |  |  |  |  |  | (0.0569) |  |
| *dQC* | 0.4881 | *** | 0.7720 | *** | 0.6619 | *** |  | 0.3444 | *** | 0.4070 | *** | 0.3738 | *** |
|  | (0.1224) |  | (0.1811) |  | (0.1689) |  |  | (0.0803) |  | (0.0831) |  | (0.0845) |  |
| *dON* | 0.5376 | *** | 0.8093 | *** | 0.7109 | *** |  | 0.3807 | *** | 0.4251 | *** | 0.3994 | *** |
|  | (0.1134) |  | (0.1737) |  | (0.1633) |  |  | (0.0721) |  | (0.0798) |  | (0.0815) |  |
| *dBC* | 0.3444 | ** | 0.8046 | *** | 0.6869 | *** |  | 0.2433 | ** | 0.4209 | *** | 0.3839 | *** |
|  | (0.1527) |  | (0.2385) |  | (0.2244) |  |  | (0.1049) |  | (0.1173) |  | (0.1197) |  |
| *dAL* | 0.5160 | ** | 1.0215 | *** | 0.8678 | ** |  | 0.3732 | ** | 0.5358 | *** | 0.4881 | *** |
|  | (0.2187) |  | (0.3714) |  | (0.3488) |  |  | (0.1536) |  | (0.1817) |  | (0.1846) |  |
| *Constant* | -4.4391 | *** | -4.7636 | *** | -4.3101 | *** |  | -3.1411 | *** | -2.5053 | *** | -2.4243 | *** |
|  | (0.3006) |  | (0.4175) |  | (0.3891) |  |  | (0.1199) |  | (0.1068) |  | (0.1184) |  |
| ***First stage****:* ***ln(PubFunding)_t-1_*** | | |  |  |  |  |  |  |  |  |  |  |  |
| *ln(nbPatCum)_t_* | -0.3186 |  |  |  |  |  |  | -0.3188 |  |  |  |  |  |
|  | (0.2242) |  |  |  |  |  |  | (0.2242) |  |  |  |  |  |
| *ln (AvgCitPerPat)_t_* |  |  | -0.5413 |  |  |  |  |  |  | -0.5329 |  |  |  |
|  |  |  | (0.7869) |  |  |  |  |  |  | (0.7862) |  |  |  |
| *[ln (AvgCitPerPat)_t_]^2^* |  |  | 0.0403 |  |  |  |  |  |  | 0.0371 |  |  |  |
|  |  |  | (0.3683) |  |  |  |  |  |  | (0.3682) |  |  |  |
| *ln (AvgClaimPerPat)_t_* |  |  |  |  | 0.0403 |  |  |  |  |  |  | -0.4024 | * |
|  |  |  |  |  | (0.3683) |  |  |  |  |  |  | (0.2433) |  |
| *[ln (AvgClaimPerPat)_t_]^2^* |  |  |  |  | 0.2141 | *** |  |  |  |  |  | 0.2134 | *** |
|  |  |  |  |  | (0.0724) |  |  |  |  |  |  | (0.0724) |  |
| *dQC* | -2.2132 | *** | -2.2964 | *** | -2.2538 | *** |  | -2.2102 | *** | -2.2869 | *** | -2.2455 | *** |
|  | (0.3695) |  | (0.3709) |  | (0.3713) |  |  | (0.3695) |  | (0.3708) |  | (0.3711) |  |
| *dON* | -2.3169 | *** | -2.3967 | *** | -2.3683 | *** |  | -2.3132 | *** | -2.3868 | *** | -2.3596 | *** |
|  | (0.3624) |  | (0.3623) |  | (0.3640) |  |  | (0.3625) |  | (0.3625) |  | (0.3640) |  |
| *dBC* | -2.5050 | *** | -2.6233 | *** | -2.5932 | *** |  | -2.5009 | *** | -2.6120 | *** | -2.5833 | *** |
|  | (0.5113) |  | (0.5087) |  | (0.5084) |  |  | (0.5112) |  | (0.5088) |  | (0.5084) |  |
| *dAL* | -3.1810 | *** | -3.2834 | *** | -3.2329 | *** |  | -3.1780 | *** | -3.2726 | *** | -3.2234 | *** |
|  | (0.7284) |  | (0.7191) |  | (0.7183) |  |  | (0.7286) |  | (0.7192) |  | (0.7184) |  |
| *dCAResearchChair_t_* | 2.6124 | *** | 2.1398 | *** | 2.2979 | *** |  | 2.6194 | *** | 2.1870 | *** | 2.3395 | *** |
|  | (0.4653) |  | (0.4655) |  | (0.4730) |  |  | (0.4655) |  | (0.4651) |  | (0.4717) |  |
| *ResearchCareerAge_t_* | 0.8746 | *** | 0.8667 | *** | 0.9083 | *** |  | 0.8700 | *** | 0.8643 | *** | 0.9060 | *** |
|  | (0.0631) |  | (0.0606) |  | (0.0642) |  |  | (0.0633) |  | (0.0608) |  | (0.0644) |  |
| *[ResearchCarerAge_t_]^2^* | -0.0260 | *** | -0.0261 | *** | -0.0274 | *** |  | -0.0257 | *** | -0.0259 | *** | -0.0273 | *** |
|  | (0.0029) |  | (0.0026) |  | (0.0028) |  |  | (0.0029) |  | (0.0027) |  | (0.0028) |  |
| *ln(nbArtCum_t_)* | -0.9626 | *** | -0.8099 | *** | -0.8707 | *** |  | -0.9599 | *** | -0.8289 | *** | -0.8877 | *** |
|  | (0.2699) |  | (0.2621) |  | (0.2705) |  |  | (0.2715) |  | (0.2627) |  | (0.2705) |  |
| *[ln(nbArtCum_t_)]^2^* | 0.2022 | *** | 0.1802 | *** | 0.1897 | *** |  | 0.2012 | *** | 0.1837 | *** | 0.1929 | *** |
|  | (0.0729) |  | (0.0676) |  | (0.0706) |  |  | (0.0735) |  | (0.0683) |  | (0.0711) |  |
| *Constant* | 5.6065 | *** | 5.3585 | *** | 5.0668 | *** |  | 5.6131 | *** | 5.3616 | *** | 5.0708 | *** |
|  | (0.4741) |  | (0.4668) |  | (0.4796) |  |  | (0.4739) |  | (0.4657) |  | (0.4787) |  |
| *ln(α)* | -0.1420 | *** | -0.2534 | *** | -0.2151 | *** |  |  |  |  |  |  |  |
|  | (0.0237) |  | (0.0339) |  | (0.0317) |  |  |  |  |  |  |  |  |
| *Nb observations* | 7664 |  | 7664 |  | 7664 |  |  | 7664 |  | 7664 |  | 7664 |  |
| *Wald χ^2^* | 496 | *** | 114 | *** | 138 | *** |  | 538 | *** | 275 | *** | 273 | *** |
| *Log likelihood* | -24996 |  | -25271 |  | -25228 |  |  | -24601 |  | -24854 |  | -24811 |  |

Note: ***, **, * show significance at the 1%, 5% and 10% levels and standard errors are presented in parentheses. The results of the ivprobit models for this dummy variable are exactly the same as Table 1.
