# Supplementary material for: Do patents of academic funded researchers enjoy a longer life? A study of patent renewal decisions
Source: PLoS One. 2018 Aug 29;13(8):e0202643. doi: 10.1371/journal.pone.0202643 (PMC6114791; doi:10.1371/journal.pone.0202643)
Supplement: S7 Table — (DOCX) [file pone.0202643.s007.docx]

**S7 Table. Impact of government funding on 8-year patent renewal decisions (*NumPatentRenew8*) in Canada – Regression results of the ivtobit and ivprobit model**

| ***Variables*** | **ivtobit**  **dependent variable:** ***NumPatentRenew8*** | | | | | |  | **ivprobit**  **dependent variable:** ***dNumPatentRenew8*** | | | | | |
| --- | --- | --- | --- | --- | --- | --- | --- | --- | --- | --- | --- | --- | --- |
|  | **(1)** | | **(2)** | | **(3)** | |  | **(1)** | | **(2)** | | **(3)** | |
| *ln(PubFunding)_t-1_* | 0.5856 | *** | 0.6461 | *** | 0.6174 | *** |  | 0.1826 | *** | 0.1837 | *** | 0.1830 | *** |
|  | (0.0884) |  | (0.0973) |  | (0.0902) |  |  | (0.0073) |  | (0.0072) |  | (0.0072) |  |
| *ln(nbPatCum)_t_* | 0.9241 | *** |  |  |  |  |  | 0.2868 | *** |  |  |  |  |
|  | (0.1536) |  |  |  |  |  |  | (0.0562) |  |  |  |  |  |
| *ln (AvgCitPerPat)_t_* |  |  | 1.5166 | * |  |  |  |  |  | 0.4328 | * |  |  |
|  |  |  | (0.7883) |  |  |  |  |  |  | (0.2295) |  |  |  |
| *[ln (AvgCitPerPat)_t_]^2^* |  |  | -0.6662 |  |  |  |  |  |  | -0.1934 |  |  |  |
|  |  |  | (0.4825) |  |  |  |  |  |  | (0.1455) |  |  |  |
| *ln (AvgClaimPerPat)_t_* |  |  |  |  | 2.2035 | *** |  |  |  |  |  | 0.6631 | *** |
|  |  |  |  |  | (0.5114) |  |  |  |  |  |  | (0.1674) |  |
| *[ln (AvgClaimPerPat)_t_]^2^* |  |  |  |  | -1.2033 | *** |  |  |  |  |  | -0.3624 | *** |
|  |  |  |  |  | (0.2763) |  |  |  |  |  |  | (0.0916) |  |
| *dQC* | 1.5699 | *** | 1.7522 | *** | 1.6453 | *** |  | 0.4898 | *** | 0.4988 | *** | 0.4883 | *** |
|  | (0.3723) |  | (0.4189) |  | (0.3931) |  |  | (0.0864) |  | (0.0876) |  | (0.0876) |  |
| *dON* | 1.8039 | *** | 1.9361 | *** | 1.8563 | *** |  | 0.5619 | *** | 0.5503 | *** | 0.5497 | *** |
|  | (0.3550) |  | (0.3966) |  | (0.3739) |  |  | (0.0766) |  | (0.0793) |  | (0.0791) |  |
| *dBC* | 2.1078 | *** | 2.4684 | *** | 2.3390 | *** |  | 0.6647 | *** | 0.7058 | *** | 0.6975 | *** |
|  | (0.4454) |  | (0.5281) |  | (0.4903) |  |  | (0.1110) |  | (0.1202) |  | (0.1180) |  |
| *dAL* | 2.2384 | *** | 2.6884 | *** | 2.5241 | *** |  | 0.7000 | *** | 0.7614 | *** | 0.7445 | *** |
|  | (0.6558) |  | (0.7797) |  | (0.7374) |  |  | -3.2759 | *** | -2.9230 | *** | -2.9236 | *** |
| *Constant* | -10.4745 | *** | -10.2471 | *** | -9.8317 | *** |  | -3.2759 | *** | -2.9230 | *** | -2.9236 | *** |
|  | (1.0912) |  | (1.1763) |  | (1.0909) |  |  | (0.1152) |  | (0.0834) |  | (0.0832) |  |
| ***First stage****:* ***ln(PubFunding)_t-1_*** | | |  |  |  |  |  |  |  |  |  |  |  |
| *ln(nbPatCum)_t_* | -0.3223 |  |  |  |  |  |  | -0.3215 |  |  |  |  |  |
|  | (0.2233) |  |  |  |  |  |  | (0.2233) |  |  |  |  |  |
| *ln (AvgCitPerPat)_t_* |  |  | -0.4994 |  |  |  |  |  |  | -0.4937 |  |  |  |
|  |  |  | (0.7841) |  |  |  |  |  |  | (0.7837) |  |  |  |
| *[ln (AvgCitPerPat)_t_]^2^* |  |  | 0.0243 |  |  |  |  |  |  | 0.0222 |  |  |  |
|  |  |  | (0.3671) |  |  |  |  |  |  | (0.3671) |  |  |  |
| *ln (AvgClaimPerPat)_t_* |  |  |  |  | -0.3857 |  |  |  |  |  |  | -0.3837 |  |
|  |  |  |  |  | (0.2429) |  |  |  |  |  |  | (0.2427) |  |
| *[ln (AvgClaimPerPat)_t_]^2^* |  |  |  |  | 0.2086 | *** |  |  |  |  |  | 0.2080 | *** |
|  |  |  |  |  | (0.0722) |  |  |  |  |  |  | (0.0722) |  |
| *dQC* | -2.1965 | *** | -2.2383 | *** | -2.2093 | *** |  | -2.1925 | *** | -2.2318 | *** | -2.2031 | *** |
|  | (0.3639) |  | (0.3658) |  | (0.3653) |  |  | (0.3639) |  | (0.3657) |  | (0.3652) |  |
| *dON* | -2.2931 | *** | -2.3269 | *** | -2.3134 | *** |  | -2.2887 | *** | -2.3199 | *** | -2.3067 | *** |
|  | (0.3523) |  | (0.3545) |  | (0.3544) |  |  | (0.3526) |  | (0.3545) |  | (0.3544) |  |
| *dBC* | -2.4766 | *** | -2.5430 | *** | -2.5301 | *** |  | -2.4717 | *** | -2.5349 | *** | -2.5225 | *** |
|  | (0.5057) |  | (0.5040) |  | (0.5029) |  |  | (0.5058) |  | (0.5041) |  | (0.5030) |  |
| *dAL* | -3.1702 | *** | -3.2246 | *** | -3.1899 | *** |  | -3.1657 | *** | -3.2170 | *** | -3.1828 | *** |
|  | (0.7301) |  | (0.7230) |  | (0.7207) |  |  | (0.7303) |  | (0.7231) |  | (0.7209) |  |
| *dCAResearchChair_t_* | 1.7605 | *** | 1.5951 | *** | 1.6675 | *** |  | 1.7837 | *** | 1.6233 | *** | 1.6946 | *** |
|  | (0.4529) |  | (0.4558) |  | (0.4552) |  |  | (0.4549) |  | (0.4568) |  | (0.4562) |  |
| *ResearchCareerAge_t_* | 0.8769 | *** | 0.8502 | *** | 0.8987 | *** |  | 0.8747 | *** | 0.8495 | *** | 0.8981 | *** |
|  | (0.0572) |  | (0.0570) |  | (0.0599) |  |  | (0.0576) |  | (0.0572) |  | (0.0602) |  |
| *[ResearchCarerAge_t_]^2^* | -0.0259 | *** | -0.0249 | *** | -0.0266 | *** |  | -0.0258 | *** | -0.0248 | *** | -0.0266 | *** |
|  | (0.0023) |  | (0.0023) |  | (0.0024) |  |  | (0.0024) |  | (0.0023) |  | (0.0024) |  |
| *ln(nbArtCum_t_)* | -0.8832 | *** | -0.7986 | *** | -0.8410 | *** |  | -0.8890 | *** | -0.8110 | *** | -0.8525 | *** |
|  | (0.2297) |  | (0.2366) |  | (0.2399) |  |  | (0.2316) |  | (0.2371) |  | (0.2404) |  |
| *[ln(nbArtCum_t_)]^2^* | 0.1711 | *** | 0.1590 | *** | 0.1662 | *** |  | 0.1719 | *** | 0.1609 | *** | 0.1678 | *** |
|  | (0.0567) |  | (0.0577) |  | (0.0586) |  |  | (0.0577) |  | (0.0584) |  | (0.0593) |  |
| *Constant* | 5.5863 | *** | 5.3679 | *** | 5.0654 | *** |  | 5.5892 | *** | 5.3686 | *** | 5.0659 | *** |
|  | (0.4751) |  | (0.4644) |  | (0.4790) |  |  | (0.4748) |  | (0.4638) |  | (0.4784) |  |
| *ln(α)* | -0.5946 | *** | -0.6538 | *** | -0.6237 | *** |  |  |  |  |  |  |  |
|  | (0.0886) |  | (0.0963) |  | (0.0896) |  |  |  |  |  |  |  |  |
| *Nb observations* | 7664 |  | 7664 |  | 7664 |  |  | 7664 |  | 7664 |  | 7664 |  |
| *Wald χ^2^* | 120 | *** | 101 | *** | 116 | *** |  | 1211 | *** | 1227 | *** | 1285 | *** |
| *Log likelihood* | -23742 |  | -23810 |  | -23781 |  |  | -23604 |  | -23668 |  | -23639 |  |

Notes: ***, **, * show significance at the 1%, 5% and 10% levels and standard errors are presented in parentheses. The results of the ivprobit models for this dummy variable are exactly the same as Table 2.
