# Supplementary material for: Do patents of academic funded researchers enjoy a longer life? A study of patent renewal decisions
Source: PLoS One. 2018 Aug 29;13(8):e0202643. doi: 10.1371/journal.pone.0202643 (PMC6114791; doi:10.1371/journal.pone.0202643)
Supplement: S8 Table — (DOCX) [file pone.0202643.s008.docx]

S8 Table. Impact of government funding on 12-year patent renewal decisions (*NumPatentRenew12*) in Canada – Regression results of the ivtobit and probit model

| ***Variables*** | **ivtobit**  **dependent variable:** ***NumPatentRenew12*** | | | | | |  | **ivprobit**  **dependent variable:** ***dNumPatentRenew12*** | | | | | |
| --- | --- | --- | --- | --- | --- | --- | --- | --- | --- | --- | --- | --- | --- |
|  | **(1)** | | **(2)** | | **(3)** | |  | **(1)** | | **(2)** | | **(3)** | |
| *ln(PubFunding)_t-1_* | 0.9573 | *** | 0.9202 | *** | 0.9096 | *** |  | 0.1905 | *** | 0.1906 | *** | 0.1906 | *** |
|  | (0.2601) |  | (0.2254) |  | (0.2217) |  |  | (0.0079) |  | (0.0082) |  | (0.0082) |  |
| *ln(nbPatCum)_t_* | 0.9901 | *** |  |  |  |  |  | 0.1810 | *** |  |  |  |  |
|  | (0.2605) |  |  |  |  |  |  | (0.0598) |  |  |  |  |  |
| *ln (AvgCitPerPat)_t_* |  |  | 15.6989 | *** |  |  |  |  |  | 3.0412 | *** |  |  |
|  |  |  | (4.1994) |  |  |  |  |  |  | (1.1290) |  |  |  |
| *[ln (AvgCitPerPat)_t_]^2^* |  |  | -32.1185 | *** |  |  |  |  |  | -6.2562 | ** |  |  |
|  |  |  | (11.0251) |  |  |  |  |  |  | (2.7264) |  |  |  |
| *ln (AvgClaimPerPat)_t_* |  |  |  |  | 1.7763 | ** |  |  |  |  |  | 0.3661 | ** |
|  |  |  |  |  | (0.8404) |  |  |  |  |  |  | (0.1783) |  |
| *[ln (AvgClaimPerPat)_t_]^2^* |  |  |  |  | -0.8777 | ** |  |  |  |  |  | -0.1748 | * |
|  |  |  |  |  | (0.4268) |  |  |  |  |  |  | (0.0899) |  |
| *dQC* | 2.7123 | *** | 2.6545 | *** | 2.6080 | *** |  | 0.4847 | *** | 0.4953 | *** | 0.4917 | *** |
|  | (0.8262) |  | (0.7683) |  | (0.7495) |  |  | (0.0914) |  | (0.0927) |  | (0.0924) |  |
| *dON* | 2.9203 | *** | 2.7945 | *** | 2.7761 | *** |  | 0.5237 | *** | 0.5229 | *** | 0.5254 | *** |
|  | (0.8712) |  | (0.7930) |  | (0.7775) |  |  | (0.0883) |  | (0.0910) |  | (0.0904) |  |
| *dBC* | 3.6310 | *** | 3.7516 | *** | 3.6996 | *** |  | 0.6704 | *** | 0.7198 | *** | 0.7162 | *** |
|  | (0.9997) |  | (0.9742) |  | (0.9581) |  |  | (0.1231) |  | (0.1352) |  | (0.1346) |  |
| *dAL* | 4.0431 | *** | 4.1025 | *** | 4.0338 | *** |  | 0.6704 | *** | 0.7198 | *** | 0.7162 | *** |
|  | (1.2967) |  | (1.1712) |  | (1.1570) |  |  | (0.1231) |  | (0.1352) |  | (0.1346) |  |
| *Constant* | -16.8785 | *** | -15.3809 | *** | -15.2199 | *** |  | -3.2538 | *** | -3.0918 | *** | -3.0896 | *** |
|  | (3.2022) |  | (2.6857) |  | (2.6420) |  |  | (0.2187) |  | (0.1676) |  | (0.1673) |  |
| ***First stage****:* ***ln(PubFunding)_t-1_*** | | |  |  |  |  |  |  |  |  |  |  |  |
| *ln(nbPatCum)_t_* | -0.3163 |  |  |  |  |  |  | -0.2898 |  |  |  |  |  |
|  | (0.2225) |  |  |  |  |  |  | (0.2299) |  |  |  |  |  |
| *ln (AvgCitPerPat)_t_* |  |  | -0.4187 |  |  |  |  |  |  | -0.2111 |  |  |  |
|  |  |  | (0.7808) |  |  |  |  |  |  | (0.8811) |  |  |  |
| *[ln (AvgCitPerPat)_t_]^2^* |  |  | -0.0084 |  |  |  |  |  |  | -0.1785 |  |  |  |
|  |  |  | (0.3668) |  |  |  |  |  |  | (0.4422) |  |  |  |
| *ln (AvgClaimPerPat)_t_* |  |  |  |  | -0.3674 |  |  |  |  |  |  | -0.4891 | * |
|  |  |  |  |  | (0.2409) |  |  |  |  |  |  | (0.2537) |  |
| *[ln (AvgClaimPerPat)_t_]^2^* |  |  |  |  | 0.1998 | *** |  |  |  |  |  | 0.2249 | *** |
|  |  |  |  |  | (0.0717) |  |  |  |  |  |  | (0.0763) |  |
| *dQC* | -2.1399 | *** | -2.1536 | *** | -2.1297 | *** |  | -1.9135 | *** | -1.9288 | *** | -1.9086 | *** |
|  | (0.3641) |  | (0.3657) |  | (0.3653) |  |  | (0.3793) |  | (0.3809) |  | (0.3811) |  |
| *dON* | -2.2322 | *** | -2.2353 | *** | -2.2270 | *** |  | -1.9897 | *** | -1.9953 | *** | -1.9956 | *** |
|  | (0.3540) |  | (0.3568) |  | (0.3564) |  |  | (0.3693) |  | (0.3722) |  | (0.3719) |  |
| *dBC* | -2.4076 | *** | -2.4375 | *** | -2.4313 | *** |  | -2.2338 | *** | -2.2652 | *** | -2.2620 | *** |
|  | (0.5061) |  | (0.5048) |  | (0.5033) |  |  | (0.5430) |  | (0.5413) |  | (0.5404) |  |
| *dAL* | -3.1101 | *** | -3.1303 | *** | -3.1020 | *** |  | -2.9587 | *** | -2.9784 | *** | -2.9505 | *** |
|  | (0.7317) |  | (0.7253) |  | (0.7231) |  |  | (0.7764) |  | (0.7706) |  | (0.7708) |  |
| *dCAResearchChair_t_* | 1.7838 | *** | 1.8114 | *** | 1.8317 | *** |  | 1.6219 | *** | 1.6370 | *** | 1.6482 | *** |
|  | (0.5581) |  | (0.5487) |  | (0.5463) |  |  | (0.5777) |  | (0.5650) |  | (0.5665) |  |
| *ResearchCareerAge_t_* | 0.8244 | *** | 0.8049 | *** | 0.8535 | *** |  | 0.8263 | *** | 0.8060 | *** | 0.8435 | *** |
|  | (0.0582) |  | (0.0589) |  | (0.0615) |  |  | (0.0616) |  | (0.0620) |  | (0.0646) |  |
| *[ResearchCarerAge_t_]^2^* | -0.0230 | *** | -0.0223 | *** | -0.0241 | *** |  | -0.0230 | *** | -0.0223 | *** | -0.0237 | *** |
|  | (0.0024) |  | (0.0024) |  | (0.0025) |  |  | (0.0025) |  | (0.0025) |  | (0.0026) |  |
| *ln(nbArtCum_t_)* | -0.9390 | *** | -0.9180 | *** | -0.9517 | *** |  | -0.8069 | *** | -0.7799 | *** | -0.7994 | *** |
|  | (0.2493) |  | (0.2568) |  | (0.2592) |  |  | (0.2438) |  | (0.2522) |  | (0.2541) |  |
| *[ln(nbArtCum_t_)]^2^* | 0.1803 | *** | 0.1764 | *** | 0.1826 | *** |  | 0.1500 | ** | 0.1449 | ** | 0.1486 | ** |
|  | (0.0616) |  | (0.0644) |  | (0.0646) |  |  | (0.0594) |  | (0.0625) |  | (0.0624) |  |
| *Constant* | 5.6849 | *** | 5.4594 | *** | 5.1631 | *** |  | 5.4524 | *** | 5.2562 | *** | 5.0223 | *** |
|  | (0.4731) |  | (0.4596) |  | (0.4748) |  |  | (0.4989) |  | (0.4842) |  | (0.5010) |  |
| *ln(α)* | -0.9854 | *** | -0.9366 | *** | -0.9260 | *** |  |  |  |  |  |  |  |
|  | (0.2648) |  | (0.2270) |  | (0.2239) |  |  |  |  |  |  |  |  |
| *Nb observations* | 7664 |  | 7664 |  | 7664 |  |  | 7664 |  | 7664 |  | 7664 |  |
| *Wald χ^2^* | 550 | *** | 98 | *** | 46 | *** |  | 1197 | *** | 1830 | *** | 1289 | *** |
| *Log likelihood* | -23069 |  | -23111 |  | -23100 |  |  | -21185 |  | -21197 |  | -21191 |  |

Notes: ***, **, * show significance at the 1%, 5% and 10% levels and standard errors are presented in parentheses. The results of the ivprobit models for this dummy variable are exactly the same as Table 3.
